# Development of Aspirin-Inducible Biosensors in *Escherichia coli* and SimCells

**DOI:** 10.1128/AEM.02959-18

**Published:** 2019-03-06

**Authors:** Jack Xiaoyu Chen, Harrison Steel, Yin-Hu Wu, Yun Wang, Jiabao Xu, Cordelia P. N. Rampley, Ian P. Thompson, Antonis Papachristodoulou, Wei E. Huang

**Affiliations:** a Department of Engineering Science, University of Oxford, Oxford, United Kingdom; b Environmental Simulation and Pollution Control State Key Joint Laboratory, State Environmental Protection Key Laboratory of Microorganism Application and Risk Control (SMARC), School of Environment, Tsinghua University, Beijing, People’s Republic of China; McMaster University

**Keywords:** LysR, SimCells, aspirin, biosensors, gene regulation, synthetic biology

## Abstract

A simple aspirin-inducible system has been developed and characterized in Escherichia coli by employing the *P_sal_* promoter and SalR regulation system originally from Acinetobacter baylyi ADP1. Mutagenesis at the DNA binding domain (DBD) and chemical recognition domain (CRD) of the SalR protein in A. baylyi ADP1 suggests that the effector-free form, SalR_r_, can compete with the effector-bound form, SalR_a_, binding the *P_sal_* promoter and repressing gene transcription. The induction of the *P_sal_* promoter was compared in two different gene circuit designs: a simple regulation system (SRS) and positive autoregulation (PAR). Both regulatory circuits were induced in a dose-dependent manner in the presence of 0.05 to 10 µM aspirin. Overexpression of SalR in the SRS circuit reduced both baseline leakiness and the strength of the *P_sal_* promoter. The PAR circuit forms a positive feedback loop that fine-tunes the level of SalR. A mathematical simulation based on the SalR_r_/SalR_a_ competitive binding model not only fit the observed experimental results in SRS and PAR circuits but also predicted the performance of a new gene circuit design for which weak expression of SalR in the SRS circuit should significantly improve induction strength. The experimental result is in good agreement with this prediction, validating the SalR_r_/SalR_a_ competitive binding model. The aspirin-inducible systems were also functional in probiotic strain E. coli Nissle 1917 and SimCells produced from E. coli MC1000 *ΔminD*. These well-characterized and modularized aspirin-inducible gene circuits would be useful biobricks for synthetic biology.

**IMPORTANCE** An aspirin-inducible SalR/*P_sal_* regulation system, originally from Acinetobacter baylyi ADP1, has been designed for E. coli strains. SalR is a typical LysR-type transcriptional regulator (LTTR) family protein and activates the *P_sal_* promoter in the presence of aspirin or salicylate in the range of 0.05 to 10 µM. The experimental results and mathematical simulations support the competitive binding model of the SalR/*P_sal_* regulation system in which SalR_r_ competes with SalR_a_ to bind the *P_sal_* promoter and affect gene transcription. The competitive binding model successfully predicted that weak SalR expression would significantly improve the inducible strength of the SalR/*P_sal_* regulation system, which is confirmed by the experimental results. This provides an important mechanism model to fine-tune transcriptional regulation of the LTTR family, which is the largest family of transcriptional regulators in the prokaryotic kingdom. In addition, the SalR/*P_sal_* regulation system was also functional in probiotic strain E. coli Nissle 1917 and minicell-derived SimCells, which would be a useful biobrick for environmental and medical applications.

## Abstract (precis)

An aspirin-inducible SalR/*P_sal_* regulation system, originally from Acinetobacter baylyi ADP1, has been designed for E. coli strains. SalR is a typical LysR-type transcriptional regulator (LTTR) family protein and activates the *P_sal_* promoter in the presence of aspirin or salicylate in the range of 0.05 to 10 µM. The experimental results and mathematical simulations support the competitive binding model of the SalR/*P_sal_* regulation system in which SalR_r_ competes with SalR_a_ to bind the *P_sal_* promoter and affect gene transcription. The competitive binding model successfully predicted that weak SalR expression would significantly improve the inducible strength of the SalR/*P_sal_* regulation system, which is confirmed by the experimental results. This provides an important mechanism model to fine-tune transcriptional regulation of the LTTR family, which is the largest family of transcriptional regulators in the prokaryotic kingdom. In addition, the SalR/*P_sal_* regulation system was also functional in probiotic strain E. coli Nissle 1917 and minicell-derived SimCells, which would be a useful biobrick for environmental and medical applications.

## INTRODUCTION

Synthetic biology has the potential to engineer bacteria for disease diagnosis (1) and cancer therapy (2). Since bacteria colonize human skin, gastrointestinal tracts, and the respiratory and reproductive systems (3) and many bacteria preferentially associate with tumors (2), they are ideal agents for diagnosis and therapy. Bacterial therapy has shown great potential in biomedicine, with applications including the regulation of a host organism’s energy metabolism (4), the delivery of drugs (5), as well as modulating chemotherapy, radiotherapy, and immunotherapy treatments for cancer (6).

Advances in the utilization of feedback-based synthetic circuits for biomass yield optimization (7), spatial control of tissue regeneration (8), and bacterial diagnosis therapy (9–12) have been characterized and implemented in *in vivo* studies. An ideal bacterial design for medical applications should have the following traits. The specific bacterial chassis should be safe for human applications. The inducer should have no side effects on human health and should be able to trigger different levels of gene expression in response to different inducer concentrations. Gene circuits should have minimal cross talk and only be triggered by a specific inducer rather than molecules present naturally in the human body or in common diet. Finally, to achieve effective bacterial therapy, the release of drugs (especially cytotoxic drugs for treatment of cancer) from engineered bacteria must be accurately and reliably controllable.

Aspirin is a safe and specific inducer that has been used widely as an analgesic and anti-inflammatory drug since 1897 (13); as such, it is ideal for human applications. The clinical safe dosage of aspirin for an adult is 75 to 300 mg per day, and therapeutic blood concentration for adults is between 111 and 555 µM, whereas the toxic concentration is between 832 and 1,665 µM (14). The biological half-life of aspirin is 2 to 3 h for low dosages and 15 to 30 h for large dosages (15). Salicylate (SA) and aspirin regulatory module *nahR/P_sal_*::*xylS2* is a *Pseudomonas* species-derived gene circuit and has been applied in regulated expression of *Salmonella* species genes at the millimolar level (9).

In this study, a simple and sensitive aspirin/salicylate-regulated SalR/*P_sal_* system in Acinetobacter baylyi ADP1 has been characterized and developed to be functional in various Escherichia coli strains (16–18). As a member of the LysR-type transcriptional regulator (LTTR) superfamily, SalR controls the salicylate degradation pathways in A. baylyi ADP1 (16) and is the activator of its own promoter, *P_sal_* (16, 18). It has been shown that SalR/*P_sal_* regulation can be activated by aspirin in A. baylyi (18). We examined the mechanism of the SalR-regulated promoter *P_sal_*, characterized this system’s modularity, and demonstrate that this system functions with different gene circuit designs. Furthermore, we hypothesized that the effector-free form, SalR_r_, could compete with the effector-bound form, SalR_a_, and developed a novel mathematical model to describe the process. We designed three aspirin-responsible *P_sal_* and *salR* gene circuits, including a positive autoregulation (PAR) circuit and two simple regulation system (SRS) circuits with various promoter strengths. The performance of *P_sal_* and *salR* gene circuits has been characterized in E. coli DH5α, probiotic E. coli Nissle 1917 (19), and chromosome-free SimCells (20). A novel mathematical model quantitatively fits the experimental results, and it predicted the performance of a new gene circuit design in which the weak expression of SalR in the SRS circuit should significantly improve the induction strength. The experimental result is in good agreement with this prediction, validating the SalR_r_/SalR_a_ competitive binding model. The sensitive aspirin gene circuits were functional in both E. coli Nissle 1917 and chromosome-free SimCells produced from E. coli MC1000 *ΔminD*.

## RESULTS

### Performance of PAR and SRS circuits in E. coli DH5a.

**FIG 1:**
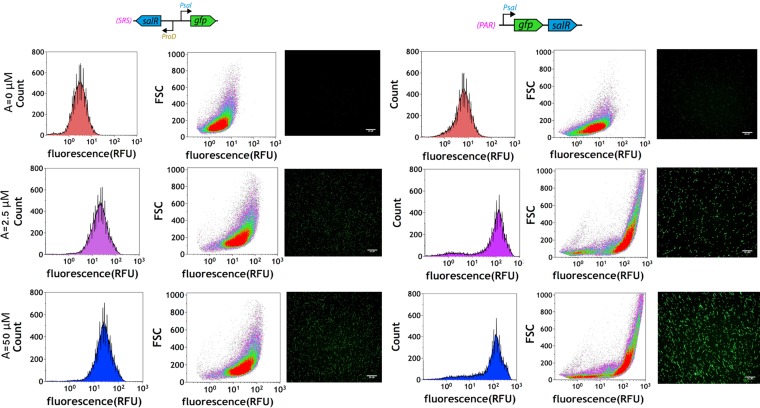
Induction of E. coli with SRS and PAR circuits in response to aspirin was evaluated by flow cytometry. The populations of more than 100,000 cells from uninduced (red), 2.5 µM aspirin (magenta), and 50 µM aspirin (blue) treatments were observed for each SRS and PAR circuit. The circuit configurations are illustrated at the top. Histograms are plotted on a logarithmic scale to render the wide range of biosensor activation.

In this study, we initially optimized codons of the *salR* gene and designed two gene circuits, a simple regulation system (SRS) and positive autoregulation (PAR) circuits, and expressed them in E. coli DH5a (Fig. 1). In the SRS circuit, the expression of *salR* is under the control of a strong constitutive promoter, *proD*, with good insulation properties in different genetic contexts (21). In the PAR circuit, *salR* is controlled by its own promoter to form a fine-tuned feedback regulation loop. E. coli DH5a organisms carrying SRS and PAR gene circuits were induced by 0, 2.5, and 50 µM aspirin, and green fluorescent protein (GFP) expression was measured at the single-cell level using flow cytometry and a fluorescence microscope (Fig. 1; see also Fig. S1 in the supplemental material). With the strong *proD* promoter of the *salR* gene, SRS has a lower background leakiness than PAR. GFP expression in both SRS and PAR can be significantly induced by 2.5 µM aspirin (Fig. 1). Density plots of front scatter (FSC) reads against the GFP channel show a single population of GFP-expressing cells in SRS at each inducing concentration, ranging from 3.67 ± 0.12 to 32.11 ± 1.45 GFP RFU (relative fluorescent units) (Fig. 1). The baseline reading in the PAR circuit was slightly higher (7.84 ± 0.82 GFP RFU) than that of the SRS circuit (3.67 ± 0.12 RFU). Furthermore, the PAR circuit exhibits a slightly bistable distribution (Fig. 1 and Fig. S1), showing two small and large populations of GFP-expressing cells, ranging from 9.69 ± 0.76 (low) to 165.16 ± 5.52 (high) GFP RFU (Fig. 1). The leaky and bistable expression of GFP in the PAR circuit is consistent with the classical mathematic models of positive autoregulation circuits (22), confirming that the synthetic PAR and SRS circuits behave as their original designs intended.

### The effector-free form, SalR_r_, is able to bind *P_sal_* promoter to repress transcription.

The aspirin-inducible regulatory system is controlled by the SalR regulatory protein, originally from Acinetobacter baylyi ADP1 (18). SalR belongs to an LTTR superfamily, the largest family of regulators in prokaryotes (23), which contains an N-end DNA binding domain (DBD) and a C-end chemical recognition domain (CRD) (24). Based on previous reports of characteristic features in the LTTR family (25), the sliding dimer mechanism was adopted and is illustrated in Fig. S2. This SalR/*P_sal_* regulation was validated using a salicylate biosensor, ADPWH_*lux,* that was previously constructed by inserting a promoterless *luxCDABE* cassette between *salA* and *salR* of the *sal* operon in A. baylyi ADP1 (18, 25).

To examine the molecular mechanism, two SalR mutants, ADPWH_NΔ*salR* and ADPWH_CΔ*salR*, were constructed with N end and C end, respectively, disrupted from ADPWH_*lux* (Table 1). In the mutant ADPWH_CΔ*salR*, four bases (ATAA) in the CRD domain of the *salR* gene were deleted using the *sacB-km* counterselection method (see Section S1). We hypothesized that this deletion did not prevent the mutated SalR from binding the *P_sal_* promoter, because the N terminus was unaltered. In the case of ADPWH_NΔ*salR*, the DBD of the *salR* gene in ADPWH_*lux* was knocked out by inserting a kanamycin resistance gene (18) into the ClaI cutting site. Hence, this ADPWH_NΔ*salR* mutant has completely disrupted the DNA binding motif of SalR and is unable to bind the *P_sal_* promoter.

**TABLE 1:** Bacterial strains and plasmids used in this study

Strain or plasmid	Description	Reference or source
Strains		
E. coli DH5α	F^–^ Φ80*lacZΔM15* Δ(*lacZYA-argF*) *U169 recA1 endA1 hsdR17* (r_K_^–^, m_K_^+^) *phoA sup*E44 λ^–^ *thi-1 gyrA96 relA1*	Laboratory collection
E. coli Nissle 1917	Ardeypharm GmbH (Herdecke, Germany)	19
E. coli MC1000 *ΔminD::km*	MC1000 strain with chromosome deletion of *minD* gene; kanamycin resistance included for selection	20
E. coli MC1000 Δ*minD*::*km* with plasmid PAR_Amp*	E. coli MC1000 Δ*minD* hosted with positive autoregulated plasmid PAR_Amp* (described below); parent cells for SimCell production	This study
ADPWH_*lux*	Closed loop autoregulated system; *luxCDABE* is inserted between *salA* and *salR* under control of *P_sal_*	18
ADPWH_NΔ*salR*	Mutant of ADPWH_*lux*, absence of four bases (ATAA) encoded for residue 195 Ile of *salR*	This study
ADPWH_CΔ*salR*	Mutant of ADPWH_*lux*, partial DBD with kanamycin insertion	This study
Plasmids		
Psal-gfp-salR (PAR)	Closed-loop autoregulated system; both *gfp* and *salR* are under control of *P_sal_*; the plasmid backbone is pMK from GeneArt cloning vector (ThermoFisher, UK)	This study
PproD-salR-Psal-gfp (SRS)	Open-loop simple expression system; *gfp* is under control of *P_sal_*; the promoter for *salR* is *Prod*; the plasmid backbone is pMK-RQ from GeneArt cloning vector (ThermoFisher, UK)	This study
PAR_Amp*	Kanamycin gene in Psal-gfp-saR (PAR) plasmid is replaced with ampicillin resistance; truncated *kan* ORF	This study
PJ109-salR-Psal-gfp (SRS*)	Open-loop simple expression system; *gfp* is under control of *P_sal_*, and *salR* is under promoter *J109*; the plasmid backbone is pMK from GeneArt cloning vector (ThermoFisher, UK)	This study
PJ109-gfp	Promoter *J109* controls *gfp*; the plasmid backbone is pMK-RQ from GeneArt cloning vector (ThermoFisher, UK)	This study
PJ115-gfp	Promoter *J115* controls *gfp*; the plasmid backbone is pMK-RQ from GeneArt cloning vector (ThermoFisher, UK)	This study
PJ106-gfp	Promoter *J106* controls *gfp*; the plasmid backbone is pMK-RQ from GeneArt cloning vector (ThermoFisher, UK)	This study

Although both SalR mutants ADPWH_NΔ*salR* and ADPWH_CΔ*salR* were unable to respond to salicylate acid, the background levels of bioluminescence expression were different (Fig. S3): the average background bioluminescence expression of ADPWH_NΔsalR was 5,968 ± 48, which is significantly higher than that of both ADPWH_CΔ*salR* (3,222 ± 66; *P* < 0.001) and ADPWH_*lux* (3,857 ± 99; *P* < 0.001). ADPWH_CΔ*salR* with a mutated C-end chemical recognition domain (but unchanged N-end DNA binding domain) should bind and repress the *P_sal_* promoter, reducing the background expression of bioluminescence, similar to ADPWH_*lux*, which has an intact *salR*. In contrast, disruption at the N end in ADPWH_NΔ*salR* completely alters the SalR structure, making it unable to bind the *P_sal_* promoter and leading to a higher background expression of bioluminescence (Fig. S3). Collectively, the results suggest that uninduced SalR or the effector-free form, SalR_r_, should bind and repress the *P_sal_* promoter.

### SalR_r_/SalR_a_ competitive binding hypothesis and mathematic model construction.

**FIG 2:**
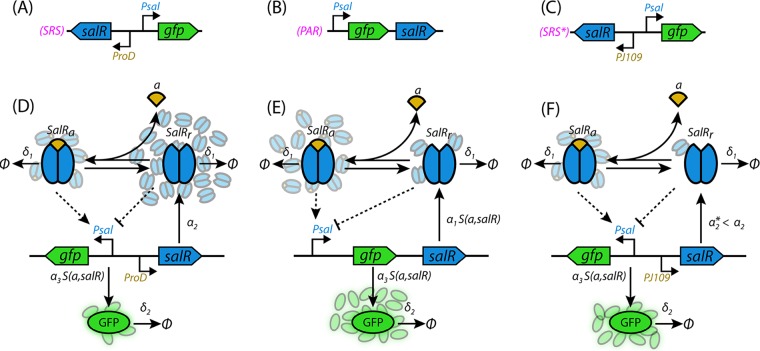
SRS, SRS*, and PAR circuits and model structure. (A) The simple regulatory system (SRS) provides open-loop and *P_sal_* promoter-controlled GFP expression, regulated by SalR under a constitutive and strong promoter, *proD*. (B) The PAR circuit closes the loop, and *P_sal_* promoter controls both *gfp* and *salR*. Initially due to transcriptional leakage, a small amount of SalR is produced, binding and repressing the *P_sal_* promoter. When the inducer is added, transcription from *P_sal_* is activated, leading to the expression of both *gfp* and *salR*. SalR binding inducers will strengthen the *P_sal_* promoter and form a loop. SalR expression is dependent on the concentrations of the inducers. (C) The simple regulatory system (SRS*) variant in which the strong promoter *proD* was replaced by a weak promoter, *PJ109*. (D) Model structure for the SRS circuit. (E) Model structure for the PAR circuit. (F) Model structure for the SRS* circuit. *gfp* is marked as green and *salR* as blue. *salR* is assumed to form dimer binding to *P_sal_* with high affinity. An inducer molecule (a, yellow) reversibly binds with the effector-free form of SalR dimer (SalR_r_) to yield its effector-bound active form (SalR_a_). SalR_a_ dimers can cooperatively bind *P_sal_* to activate gene expression, while cooperative binding of two SalR_r_ dimers can repress gene expression. For detailed model description and definition of parameters, see Information Section S1 in the supplemental material.

Accorrding to the *salR* gene mutagenesis results described above, we hypothesize that both SalR_r_ and SalR_a_ can competitively bind the *P_sal_* promoter. To assess the hypothesized mechanism and investigate how variation of different aspects of the SRS and PAR circuits affect the aspirin-regulated performance, we developed a mathematical model to simulate and predict the performance of the three gene circuits. The interaction considered in our model for the SRS and PAR systems is outlined in Fig. 2 and is implemented using deterministic differential equations and binding models which describe gene regulation and expression. This process treats the SalR regulatory mechanism as a biased competition between binding of SalR_a_, which activates the *P_sal_* promoter, and SalR_r,_ which occupies the binding site and represses the *P_sal_* promoter. For the SRS circuit, the competition between overexpressed SalR_r_ and aspirin-activated SalR_a_ is expected to produce low GFP expression levels. This competition has little effect on the PAR circuit, as its SalR level is tuned by a self-regulated feedback loop.

We created a two-state differential equation model of the system, in which the concentration of SalR is governed by

(1) $$\frac{d\;\left[salR\right]}{dt}=S{\left(\left[a\right],\left[salR\right]\right)}_{1}-\delta_{1}\left[salR\right]$$

for the PAR circuit and

(2) $$\frac{d\;\left[salR\right]}{dt}=\alpha_{2}-\delta_{1}\left[salR\right]$$

for the SRS circuit, and the circuit output (GFP) concentration is governed by (for both circuits)

(3) $$\frac{d\;\left[salR\right]}{dt}=S\left(\left[a\right],\left[salR\right]\right)\alpha_{3}-\delta_{2}\left[gfp\right]$$

where the regulating function that describes the interactions between SalR and inducer concentrations and the *P_sal_* promoter is given by

(4) $$S\left(\left[a\right],\left[salR\right]\right)=\frac{K_{R}}{K_{R}+\left[salR\right]}\left(L+\left(1-L\right)\frac{\left[salR\right]}{\left[salR\right]+K_{A}}\frac{{\left[a\right]}^{n}}{{\left[a\right]}^{n}+K_{a}^{n}}\right)$$

in which *a* is inducer concentration, *L* is the leakage rate from the *P_sal_* promoter, *K_R_* defines the saturation point for repressive binding with *P_sal_*, *K_A_* defines the saturation point for activating SalR binding with *P_sal_*, and *K_a_* defines the saturation point for inducer interaction with the circuit. Thus, equation 3 reflects the balance we expect between repressive SalR_r_ binding and inducer-dependent activation of SalR_a_ binding. Further discussion of the modeling approach, as well as the procedure used for parameter fitting, is provided in Section S2.

### Experimental characterization and model validation in E. coli DH5a.

The performance of the SRS and PAR designs in E. coli was characterized in terms of four features: (i) expression level, (ii) response time, (iii) output dynamic range/induction fold, and (iv) input dynamic range and leakiness (uninduced expression level). These four features are motivated by a desire to quantify a biosensor’s utility in drug delivery and *in vivo* sensing. In such an application a biosensor would ideally exhibit a high sensitivity, fast response, high induction fold change, and tightly controlled expression. To examine these criteria, we ran plate reader experiments to analyze the SRS and PAR circuits over a range of inducer concentrations and compared these data to the simulated results from the mathematical model.

**FIG 3:**
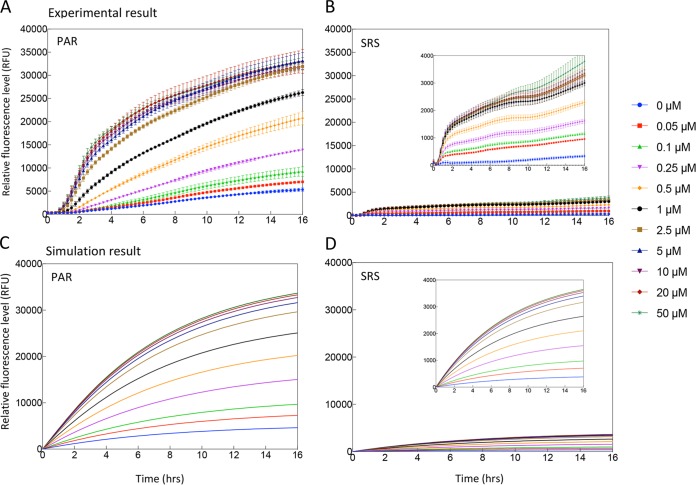
Induction kinetics for the SRS and PAR circuits in LB (A and B) and *in silico* modeling (C and D). Aspirin was added at time zero, and fluorescence was monitored every 15 min for 16 h. Color-coded induction levels are indicated on the right. The induction gradient is 0, 0.05, 0.1, 0.25, 0.5, 1, 2.5, 5, 10, 20, and 50 μM. To be easily compared with the experimental data, the simulations are plotted with the same inducer concentrations as those used for characterization. Standard deviations were plotted for all data replicates (*n* = 4).

The SRS and PAR circuits in E. coli DH5a were induced by different concentrations of aspirin, and the response time of GFP expression was examined in three different growth media (Luria-Bertani [LB] medium, phosphate-buffered saline [PBS] with arabinose, and PBS with glycerol) (Fig. 3 and Fig. S4 and S5). The resulting expression profiles represent a broad range of sensor behaviors that might arise from different external disturbances and internal cell state variation. We defined the time required for biosensor induction response in each condition as the time taken for GFP fluorescence to reach and exceed 10% of its final maximum fluorescence output.

The expression profile for both the SRS and PAR circuits in LB media is shown in Fig. 3A and B. The fluorescence and optical density (OD) were monitored for 16 h over a range of aspirin concentrations between 0.05 and 50 µM. GFP expression in both SRS and PAR circuits was detectable within 50 min, which is close to the theoretical minimum time required for GFP maturation (26). No significant difference in growth rate was observed between the SRS and PAR circuits (Fig. S6). Experimental results from the expression of PAR and SRS circuits are in good agreement with the mathematical model simulation (Fig. 3); in both cases the PAR circuit exhibited substantially higher GFP expression than the SRS circuit. In the SRS circuit, the abundant SalR_r_ competitively occupied and repressed the *P_sal_* promoter, reducing the possibility of SalR_a_ binding, which repressed overall GFP expression in the presence of aspirin and lowered the baseline level. The threshold concentration of aspirin required to activate GFP expression in the SRS circuit was 0.05 µM, and leaky expression was low (Fig. 3). The PAR circuit tuned the strength of SalR expression through a positive feedback loop and reduced the competitive repression, resulting in much stronger GFP expression with an activation threshold concentration of 0.05 μM (Fig. 3). However, the level of leaky expression of GFP in the PAR circuit was higher than that in the SRS circuit.

### Redesigned SRS* genetic circuit validates competitive binding hypothesis.

**FIG 4:**
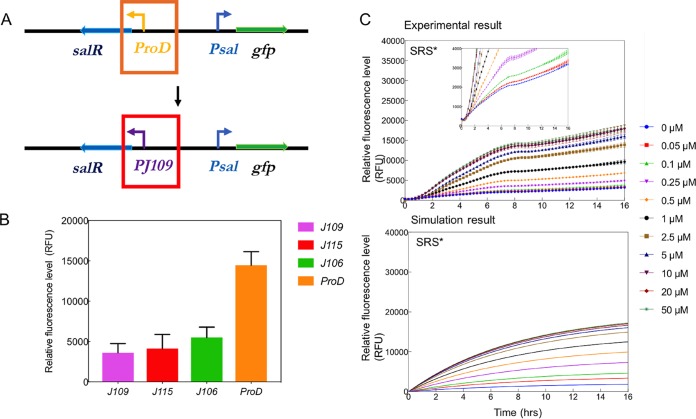
Induction kinetics for the SRS* circuit in LB and *in silico* modeling. (A) Schematic diagram of promoter replacement and the configuration of SRS*. (B) The relative fluorescence level was characterized with GFP and *J109, J115, J106,* and *proD* promoters in DH5α. (C) Aspirin was added at time zero, and fluorescence was monitored every 15 min for 16 h. Color-coded induction levels are indicated on the right. The induction gradient is 0, 0.05, 0.1, 0.25, 0.5, 1, 2.5, 5, 10, 20, and 50 µM. To be easily compared with the experimental data, the simulations are plotted with the same inducer concentrations as those used for characterization. Standard deviations were plot for all data replicates (*n* = 3). The inset figure provides a clear visualization of the induction threshold. GFP reads for inducers with 0 and 0.05 µM at the end of 16 h were calculated. *P* values between control (0 µM) and induction (0.05 µM) conditions were <0.05.

To redesign the SRS circuit, the expression of *salR* under the control of the constitutive promoter with different strengths was examined. Promoter *proD,* along with three weaker promoters, *J109*, *J115,* and *J106*, were fused with the *gfp* gene separately, and the strength was evaluated by detecting GFP expression levels. Figure 4A shows that *proD* was the strongest promoter and *J109* was the weakest promoter among the four promoters *proD, J109*, *J115,* and *J106*, which is consistent with previous reports (21, 27). Subsequently the *J109* promoter (5 times weaker than *proD*) was characterized and replaced *proD* in the construction of SRS* (Fig. 4B). Interestingly, the GFP expression level in the SRS* circuit was 3.5-fold higher than that in the original SRS circuit, and the new SRS* showed an intermediate leakiness of GFP expression compared to the SRS circuit (Fig. 3B and 4C). The threshold concentration for the SRS* circuit remained 0.05 µM (*P* < 0.05). This is in a good agreement with the competitive binding hypothesis, suggesting that the SRS* circuit with the weak promoter *J109* should produce less SalR than the SRS circuit, ease the SalR_r_/SalR_a_ competitive binding on the *P_sal_* promoter, and increase the GFP expression level (Fig. 4C). By applying the same parameters, the mathematical model fit the experimental results well and predicted that the transcriptional rate from *J109* is ∼15% of that from *proD*, similar to the output ratios observed in Fig. 4B. Collectively, the *salR* mutagenesis experiment, the behavior of the SRS* circuit, and mathematic model fitting suggest that the hypothesis of SalR_r_/SalR_a_ competitive binding is reasonable.

### Dynamic range and leakiness.

**FIG 5:**
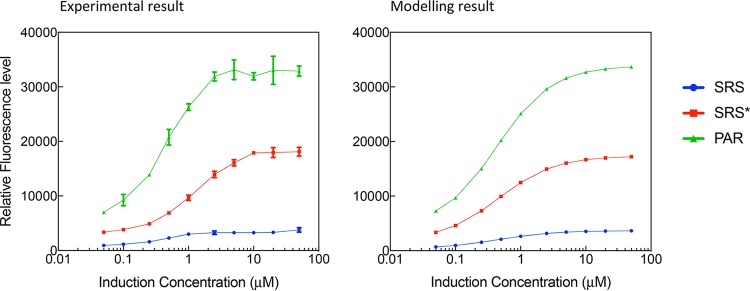
Dose-response curve for the SRS, SRS*, and PAR circuits in LB. The maximum expression point was determined at the end of the 16-h induction experiment. The induction gradient is 0, 0.05, 0.1, 0.25, 0.5, 1, 2.5, 5, 10, 20, and 50 µM. The diagram on the left corresponds to experimental data, and that on the right is the corresponding simulation result for each condition. Standard deviations were plotted for all data replicates (*n* = 3).

The dynamic range of an inducer, also referred to as its dose response, denotes the difference between the induction level that results in the biosensor’s maximal output and the induction level at which the biosensor’s output increases significantly above the uninduced level. There are a range of metrics for quantifying this criterion, such as the fold change of inducer concentration over which the sensor goes from 10% of its maximum induction level to 90% of this level (28). Figure 5 shows that the threshold of SRS, SRS*, and PAR activation by aspirin is 0.05 µM, GFP expression increases in the range of 0.05 to 10 µM, and saturation occurs when the aspirin concentration is greater than 10 µM. The mathematical model fits well to the experimental measurements (Fig. 5) in this regard. In the LB medium experiments, SRS exhibited an induction range from 334 ± 43 to 3,790 ± 405 GFP arbitrary units (AU), SRS* showed an intermediate range from 3,201 ± 45 to 18,119 ± 794 GFP AU, and PAR exhibited an induction range from 5,325 ± 362 to 32,908 ± 939 GFP AU (Fig. 5). PAR had a much higher induced expression strength than SRS in all carbon condition cases (Fig. 3 and Fig. S4); this is in line with what we expected from classical models of autoregulation, which in many established *in silico* and *in vivo* studies has demonstrated that one of the benefits of using a positive regulation system architecture is signal amplification (29–31).

These results also confirm the conceptual model of the systems (Fig. 3), indicating that the SRS circuit with strong promotion of *salR* has little leaky expression compared to that of the PAR circuit. In all experiments of the SRS circuit, GFP expression levels in uninduced controls and blank background had no detectable difference, and no measurable leakiness was observed over 16 h, suggesting a very tight control in this design. In contrast, although the PAR circuit has a strong induced expression strength, it showed a leaky background, particularly in LB media (Fig. 3). SRS* increased the expression level to 18,119 ± 794 GFP AU from 3,790 ± 405 GFP AU in SRS, at a cost of increased leakiness from 431 ± 33 to 3,201 ± 45 GFP AU.

### Performance of SRA and PAR circuits in probiotic *E. coli* Nissle 1917.

**FIG 6:**
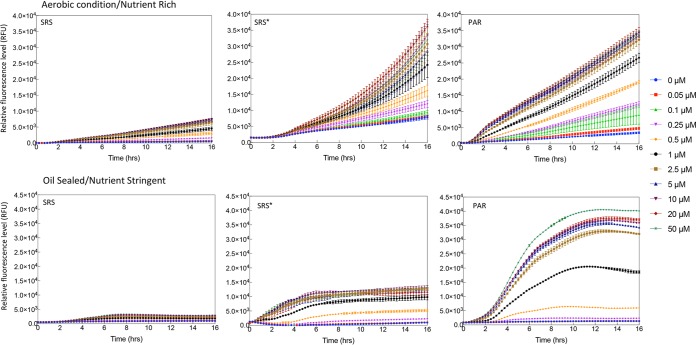
Induction kinetics for the SRS, SRS*, and PAR circuits in E. coli Nissle 1917. (Upper) The induction kinetics under aerobic conditions in nutrient rich (LB medium). (Lower) The induction kinetics under microaerobic conditions and stringent nutrient conditions (M9 medium with 0.4%, wt/vol, glucose). Different induction levels are color-coded as indicated in the legend. The induction gradient is 0, 0.05, 0.1, 0.25, 0.5, 1, 2.5, 5, 10, 20, and 50 μM.

The SRS, SRS*, and PAR circuits were then cloned into a probiotic strain, E. coli Nissle 1917. Considering that the gut environment is semiaerobic, E. coli Nissle 1917 with SRS, SRS*, and PAR circuits was characterized under both aerobic and semiaerobic conditions. To simulate the potential growth competition with host gut microbiome (32), a nutrient-limited environment was also tested. A small amount of glucose (0.4%, wt/vol) was supplied as a sole carbon source, as it is a common carbohydrate source from the human diet. E. coli Nissle 1917 with SRS, SRS*, and PAR circuits maintained a gradient response to inducer concentration under aerobic and semiaerobic conditions, highlighting the system’s modularity and robustness to a changing chassis and nutrient conditions (Fig. 6 and Fig. S6 and S7). The SRS circuit in E. coli Nissle 1917 exhibited tight control but low response to different concentrations of aspirin, while the PAR circuit demonstrated a higher response to aspirin (Fig. 6). The performance of the SRS, SRS*, and PAR circuits in E. coli Nissle 1917 was similar to that in E. coli DH5a, consistent with the hypothesis of SalR_r_/SalR_a_ competitive binding. Notably, when supplied with a limited amount of 0.4% (wt/vol) glucose, the expression profile of PAR showed less leakiness, implying that the circuit could function optimally under nutrient-limited conditions. We suspect this change is due to unknown host interference, where cells experiencing glucose starvation alter their resource allocation (33). Furthermore, we noticed there is a slight growth advantage for transformed bacteria in the case of 50 µM than for controls (0 µM) (Fig. S7).

### Aspirin is converted into salicylate in E. coli.

Based on previous work (18), it is evident that the biosensor with the SalR/*P_sal_* system can be triggered by either salicylate or aspirin. However, it is unclear if aspirin directly activates SalR or whether its metabolite, salicylate, does so. Based on the fact that cells have a slight growth advantage when supplemented with 50 µM aspirin (Fig. S7) and that E. coli has nonspecific esterase activity (34), it is possible that aspirin was metabolized into salicylate and acetate. Salicylate was the main product of aspirin degradation (35), while acetate is known to be an energy source of E. coli under stringent nutrient conditions (36). To investigate if aspirin could be cleaved by esterase to produce salicylate that then activates SalR, we used the iron(III) chloride assay (37) to analyze aspirin conversion into salicylate.

The solution of 2 mM FeCl_3_ mixing with salicylate quickly changes its color, which is detectable by absorbance analysis (Fig. S8A). Absorption curves indicate that the salicylate solution produced a peak at 300 nm after interacting with FeCl_3_ (Fig. S8B). This unique absorbance peak can be used to identify the presence of salicylate. Interestingly, a time course analysis shows that both the aspirin water control and aspirin with E. coli Nissle 1917 cells can produce salicylate (Fig. S9). This suggests that aspirin in water can be broken down to salicylate at 37°C after 3 h, although the level of salicylate was lower than that of aspirin mixed with E. coli Nissle 1917 cells (Fig. S9). It also indicates that nonspecific esterase activity in E. coli Nissle 1917 assisted aspirin degradation, as was reported previously (34).

Collectively, aspirin can be converted into salicylate nonbiologically and biologically: nonspecific esterase activity in E. coli can accelerate the aspirin degradation. Based on this result, it is likely that aspirin is being broken into salicylate which then activates the SalR regulator, although aspirin could not be completely ruled out as a direct activator.

### Performance of SRS and PAR circuits in SimCells.

**FIG 7:**
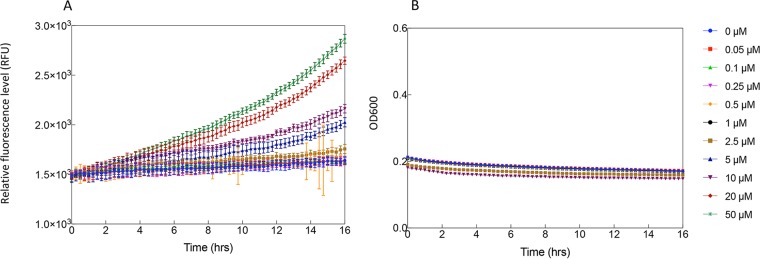
Induction kinetics for PAR circuit in SimCells. Aspirin was added at time zero, and fluorescence was observed for 16 h. (A) Induction kinetics in PBS medium. (B) Growth curves during the induction. No growth was observed, as these are chromosome-free SimCells. Induction levels are color coded as indicated in the legend. The induction gradient is 0, 0.05, 0.1, 0.25, 0.5, 1, 2.5, 5, 10, 20, and 50 μM. Standard deviations were plotted for all data replicates (*n* = 3).

The SimCells were generated from the E. coli MC1000 *ΔminD* strain with plasmid PAR_Amp* (Table 1). The PAR gene circuit was also induced and expressed in purified SimCells (Fig. 7 and Fig. S10). The lack of any growth by SimCells confirmed their lack of chromosome. Both fluorescent images (Fig. S10) and the results from the microplate reader (Fig. 7) revealed that the background leaky expression of GFP in SimCells was very low, and GFP expression in SimCells became stronger with the increase of aspirin concentrations. SimCells were usually less than 500 nm in size, and some of them were actively moving under the microscope (Fig. S10), consistent with our previous reports (20).

## DISCUSSION

### An aspirin-inducible SalR/*P_sal_* regulation system is functional in different E. coli strains and SimCells.

Probiotic bacteria are safe for human applications, but only a few can be used in gene manipulations (38, 39). E. coli Nissle 1917 is a safe probiotic bacterium frequently used for medicinal purposes (19, 40–42). Importantly, gene manipulations can be performed in E. coli Nissle 1917, making it an ideal chassis for synthetic biology. SimCells are small (400 to 600 nm), chromosome-free, and functional cells formed as a result of abnormal cell division (20). SimCells contain transcription and translation machinery, meaning they are able to faithfully express designed gene circuits without interference from background gene networks in the chromosome. Their medical utility is also aided by the fact that they are nongrowing and small. Hence, E. coli Nissle 1917 and SimCells were chosen as chassis for the expression of gene circuits in this study.

### A simple and sensitive response to aspirin in SalR/*P_sal_* system.

There is only a limited range of inducers that can effectively operate within the human body, as many inducers may adversely impact human health or are broadly found in common dietary materials and therefore should be avoided. For example, antibiotic inducers might alter the structure of microbiota in the gut, while sugar inducers may suffer from unwanted induction. However, aspirin is largely absent from common human diets, and its pharmacological safety and effectiveness have been demonstrated extensively through many clinical trials and *in vivo* studies (43, 44). For the SRS and PAR circuits studied in this work, the effective range of aspirin dosage required for activation is 0.05 to 10 µM, which is more than 10 times lower than the human safe dosage of aspirin (∼111 µM). Furthermore, induced GFP expression in cells with SRS and PAR circuits was observed to start after about 50 min and reached significant strength within 16 h (Fig. 3 and 4), which is a time scale comparable to that of aspirin’s biological half-life.

An aspirin/salicylate-triggered *nahR* regulatory system was previously developed by Royo et al. (9). A *Salmonella* species was engineered for *in vivo* expression of 5-fluorocytosine to treat mice with fibrosarcoma. This regulatory circuit is a salicylate-inducible cascade expression system derived from a naphthalene degradative pathway in Pseudomonas putida (9). It demonstrated effective drug delivery to minimize tumor size in mice, proving the concept of using salicylate/aspirin as inducers to trigger synthesis of cytosine deaminase that catalyzed production of potent cytotoxic fluorouracil for cancer chemotherapy. In this case, the gene circuit configuration was a simple cascade expression without feedback, and the effective induction concentration of aspirin was 125 to 5,000 μM (9). Given that aspirin shows toxic effects at concentrations greater than 832 μM (14), sensitive gene circuits for aspirin have an advantage in clinical practice. In addition, the well-characterized SRS and PAR circuits are simpler gene circuits than the regulatory module *nahR/P_sal_*::*xylS2*, demonstrating a dose response to a lower range of aspirin concentrations, 0.05 to 10 µM. The importance of feedback loops in genetic circuits is evident in their abundance in naturally occurring gene regulatory systems (22). As a universal network motif in transcription interactions (22, 45), feedback systems can provide several advantages, including robustness despite the presence of noise (7), improved temporal response (46), and stability in changing cellular contexts (47). The present study not only compared two gene circuits, SRS (no feedback) and PAR (with feedback), but also successfully applied a mathematical model to simulate the performance according to the LTTR transcriptional regulatory mechanism (Fig. 3 and 4). This hypothesized regulatory mechanism was further supported by altering the promoter strength and creating an intermediate SRS* circuit (no feedback and with a weaker promoter).

The results from the investigation of the molecular mechanism of *salR* regulation suggest that aspirin/salicylate can be transported into E. coli and activate the gene circuits SRS and PAR. In A. baylyi ADP1, a putative protein encoded by *salD* is presumably responsible for salicylate transport across the membrane. This protein is homologous to the E. coli FadL membrane protein (16). However, it is unclear how efficiently E. coli can transport aspirin/salicylate across its membrane. In E. coli, aromatic permeant acids such as salicylate and benzoate induce a large number of low-level multidrug efflux systems, governed by the Mar operon (*marRAB*) as well as additional, unidentified mechanisms (48). Since E. coli does not have a specific transport protein for aspirin/salicylate, it is likely the efflux system will remain on after the induction; hence, we hypothesize that there is a maximum equilibrium concentration of aspirin/salicylate inside the cell cytoplasm. This hypothesis fit well with the mathematical models to successfully simulate the performance of E. coli in response to different concentrations of aspirin and growth conditions.

### The performance of the SalR/*P_sal_* system is robust under various conditions.

In the present study, a few aspects of the SalR/*P_sal_* regulatory system were investigated: its competitive inducer binding properties, response to various inducer concentrations, and behavior in E. coli given various system architectures and growth medium conditions. To tie the diversity of experimental observations together and to test the hypotheses that they inform, we built a mathematical model and demonstrated that it was able to describe and simulate all aspects of the system’s behavior. The model not only supports the mechanism that we proposed to underlie the performance of both the PAR and SRS circuits but also provides a useful tool for the development of different systems that employ the SalR/*P_sal_* (or similar) regulatory system. These may include synthetic biological devices designed for clinical applications in the future; in such cases *a priori* utilization of the modeling architecture developed here can inform design choices and experimental approaches, thereby optimizing designs prior to their implementation.

Since many genera of bacteria naturally coexist with tumors in clinical situations (2), probiotic bacteria such as E. coli Nissle 1917 and nongrowing SimCells should be ideal chassis for the construction of anticancer agents due to their designable features, safe nonproliferation properties, and small size. Controllable expression of gene circuits, combined with safe delivery and tissue penetration, would enable E. coli Nissle 1917 and SimCells to selectively exert cytotoxicity in tumors. This study demonstrates that the aspirin-inducible SalR/*P_sal_* system is able to function in E. coli Nissle 1917 and SimCells, which would be useful for bacterial diagnosis and therapy in medicine.

## MATERIALS AND METHODS

### Chemical and reagents.

All reagents were purchased from Sigma-Aldrich (Dorset, UK) unless otherwise noted. Antibiotics (kanamycin and ampicillin) were obtained from Fisher Scientific (UK). Sterile 1× PBS solution was made by diluting 10× PBS stock solution purchased from Fisher Scientific (UK).

### Strains and plasmids.

The strains and plasmids used in this study are listed in Table 1.

Plasmids Psal-gfp-salR (PAR circuit) and PproD-salR-Psal-gfp (SRS circuit) were synthesized by GeneArts (ThermoFisher, UK). PJ109-salR-Psal-gfp was from PproD-salR-Psal-gfp, in which the *proD* promoter was replaced with the J109 promoter. PAR_Amp* was from Psal-gfp-salR, in which the kanamycin gene was replaced with an ampicillin resistance gene. Other plasmids listed in Table 1 were constructed using standard molecular cloning methods. The plasmids were transformed into E. coli DH5α cells by heat shock. E. coli Nissle 1917 was acquired from Ardeypharm GmbH (Herdecke, Germany). E. coli MC1000 Δ*minD* cells were made chemically competent before transformation with PAR_Amp*. The strains were cultured in LB medium with 50 μg·ml^−1^ kanamycin or 100 μg·ml^−1^ ampicillin (as appropriate) in a 37°C incubator.

### Gene circuit design.

The designs of both the positive autoregulation and simple regulation system plasmids employed identical modular components: *P_sal_* promoter, *gfp,* and *colE1* origin of replication (Table 1).

The codon sequence of *salR* was optimized to fit E. coli. The *salR* binding domain and *P_sal_* promoter were determined by analyzing the sequences of the *sal* operon (GenBank accession no. AF150928). SRS and PRS circuits were designed, and their structures are shown in Fig. 2A and B. In the PAR circuit, the *gfp* gene was fused directly to the *P_sal_* promoter to replace the sequence of the original *salA*, codon-optimized *salR* replaced the original *salR*, and the original structure, promoter, and ribosome binding site were kept intact in the *salR* operon. In the SRS circuit, gene-optimized *salR* is under the control of a constitutive promoter, *proD* (21), whose structure contained promoter-TACTAGAG-B0032-TACTAG-ORF-TACTAGAG-B0015, where the ORF was the *salR* gene and B0032 and B0015 are standard ribosome binding sites and terminators from the Registry of Standard Biological Parts (http://parts.igem.org/Main_Page). The full sequences of SRS and PAR were synthesized and supplied by GeneArt gene synthesis (ThermoFisher Scientific, Ltd.). Further detailed information of gene circuit design can be found in Information Section S1 in the supplemental material.

### Culture and purification of SimCells.

Due to the selection marker on E. coli MC1000 *ΔminD*::*km*, a plasmid of PAR (Psal-gfp-salR) with alternative ampicillin resistance (PAR_Amp*) was needed and was constructed as follows. The ampicillin resistance gene was isolated by PCR from the plasmid pGEM-T (Promega, UK) by using an In-Fusion cloning kit (CloneTech, UK) and the primers PAR_Amp^R^ For and Rev (Table 2). The resulting sticky-end PCR product contained an overhanging EagI site and was thereby ligated in a linearized PAR plasmid. The targeting PAR plasmid was linearized and digested with EagI-HF (New England BioLabs), which disrupted the Kan ORF. Only upon correct insertion was ampicillin resistance replaced.

**TABLE 2:** Primers used in this study

Primer	Description	Sequence (5′→3′)
Fragment PAR_Amp^R^ REV	Construction of ampicillin resistance PAR circuits	CACGCCCAGACGGCCCGCGGAACCCCTATTTGTTTATTTTTCT
Fragment PAR_Amp^R^ FOR		TTATTGATTGCGGCCTTACCAATGCTTAATCAGTGAGGCACC
ProD_Fragment_For	Construction of ProD-GFP for characterization purposes	TTAATCTCTAGTACTCTAGTAAAAGTTAAACAAAATTATTTGTAGAGGGAAACC
ProD_Fragment_Rev		GCCGCTTCTAGAGTTCTAGAGCACAGCTAACACC
ProD_Vector_For		ACTTTTACTAGAGTACTAGAGATTAAAGAGGAGAAATACTAGATGCGTAAAGGAGAAG
ProD_Vector_Rev		CTGTGCTCTAGAACTCTAGAAGCGGCCGC
J109_Promoter_For	Replacement of J109 promoter	ACTGAGCTAGCTGTAAACTTCAAAATTTAAATCTAAATATCAATGTTTTAAGATCATAATGATGG
J109_Promoter_Rev		CCTAGGGACTGTGCTAGCTCACACAGGAAAGTACTAGATGGACCTGTC
QC_SRS_For	Primers used for sequencing check of SRS	CAATTAATTACAGAACAGAGATAACTTTT
QC_SRS_Rev		GTCTCCCTGAATATATTATACGAG
QC_PAR_For	Primer used for sequencing check of PAR	CCGCATCTTCAAAGATCTAATTTA
QC_PAR_Rev		CTTTTGAAAGATCCCAACGAA
SalR_End_Rev	Construction of *Acinetobacter* ADPWH_NΔ*salR*	GCCCTCAGGAATTGGCGACTA
LuxE_For		TGGTTTACCAGTAGCGGCACG
SaR_Flank_rev	Construction of *Acinetobacter* ADPWH_CΔ*salR*	CTCTAGCAGCCGATATCGTACG
SalR1_For		CATGGAAGATTCTAAAACGTGGAC
SalR_BglII_for	Creation of BglII site for *salR* gene	CGAGATCTCTACCGGGCATACTCAGGTC
SalR_BglII_Rev		GACCTGAGTATGCCCGGTAGAGATCTCG

Following heat shock, E. coli MC1000 *ΔminD*::*km* transformants with plasmid PAR_Amp* were selected on LB agar selection plates containing 100 µg/ml ampicillin. A single colony from each plate was cultured at 37°C with continuous shaking at 200 rpm overnight in 5 ml LB broth supplemented with 100 µg/ml ampicillin (LB amp). Overnight culture was added to fresh LB-ampicillin at a ratio of 1:1,000 and cultured for 24 h. For the minicell purification, a modification of a previously described method (20) was used in order to obtain a high yield and purity while maintaining maximum ATP within the minicells. Overnight culture was centrifuged at 4°C at increasing speeds, with increments of 1,000 × *g*, from 1,000 × *g* to 4,000 × *g* for 10 min at each step to remove parent cells from the suspension. The supernatant was subsequently treated with 100 µg/ml ceftriaxone and incubated at 37°C for 1 h with shaking at 200 rpm and then stored at 4°C overnight. Following further centrifugation at 4,000 × *g* for 15 min to pellet any remaining lysed and elongated parent cells as a result of the ceftriaxone treatment, the supernatant was passed through a 0.22-µm nitrocellulose membrane (Sigma, UK) and the minicells resuspended from the membrane into sterile PBS solution to concentrate them 100-fold. PBS was chosen as it is a cost-effective medium in which to suspend the cells and to maintain osmotic balance. Minicell samples were maintained at 4°C prior to testing. The purity of minicell suspensions was determined by a plate count method after 24 h of incubation on LB-ampicillin agar plates at 37°C and conducted in triplicate.

### Flow cytometry.

Transformed E. coli DH5α cells were induced with an aspirin concentration gradient (final concentrations of 0, 0.05, 0.1, 0.25, 0.5, 1, 2.5, 5, 10, 20, and 50 μM) in kanamycin (50 µM) and LB for 16 h at 37°C in a 600-rpm shaker. Induced culture was then analyzed using a FACSCalibur (BD Bioscience), with 100,000 events being captured for each sample. Gating was performed on forward and side scatter and the GFP channel to remove noise caused by debris and background. Data were exported to Kaluza flow cytometry for subsequent analysis and visualization.

### Induction and GFP measurements.

A stock solution of l-arabinose, glycerol, and aspirin was made in deionized water and sterilized using a 0.2-μm syringe filter to be tested with cells containing SRS and PAR circuits. A total volume of 200 μl (cultured bacterial cells and inducing aspirin) was loaded in a 96-well black-sided, clear-bottomed plate (Nunclon, UK) in quadruplicate. The plate was then placed into a BioTek Synergy HT microplate reader (BioTek Corporation, UK) maintained at 37°C with reads of fluorescence (excitation, 480 nm; emission, 520 nm) and OD at 600 nm. Readings were recorded every 15 min for 16 h. Cellular intrinsic fluorescence background was subtracted from the control of E. coli without plasmid.

For experimental conditions in PBS and M9 prior to induction, transformed bacteria, which were cultured overnight in LB, were centrifuged and washed with PBS and M9 three times at 4°C to remove any residue of LB broth. For achieving microaerobic conditions, 50 μl of mineral oil was added on top of the 200-μl reaction volume in a 96-well plate.

### Visualization of bacterial cells.

Fluorescence of induced bacterial cells or SimCells was visualized using a Motica BA210 digital microscope with Moticam 580INT display output after 16 h of induction. Five μl of each sample was taken from the well and placed on a glass slide with a coverslip. Images were taken at ×100 magnification. Light micrographs were subsequently analyzed using ImageJ, version 1.50 b, with fluorescent maxima automatically counted after consistent thresholding.

### FeCl_3_ assay of measuring aspirin conversion to salicylate.

To study aspirin conversion to salicylate, E. coli Nissle 1917 culture with 5 mM aspirin was incubated at 37°C and sampled at 0, 3, and 16 h. The samples were centrifuged at 5,000 × *g* for 5 min to remove cell pellets and only the supernatants were used for FeCl_3_ assay, as dictated by the protocol (37). FeCl_3_ stock solution was prepared using FeCl_3_ anhydrous powder. Absorbance scans were done using a Spark multimode microplate reader (Tecan Ltd., UK).

## Supplementary Material

Supplemental file 1

## References

[1] Slomovic S, Pardee K, Collins JJ 2015 Synthetic biology devices for in vitro and in vivo diagnostics. Proc Natl Acad Sci U S A 112:14429–14435. doi:10.1073/pnas.1508521112. 26598662 PMC4664311

[2] Forbes NS 2010 Engineering the perfect (bacterial) cancer therapy. Nat Rev Cancer 10:785–794. doi:10.1038/nrc2934. 20944664 PMC3756932

[3] Sender R, Fuchs S, Milo R 2016 Revised estimates for the number of human and bacteria cells in the body. PLoS Biol 14:e1002533. doi:10.1371/journal.pbio.1002533. 27541692 PMC4991899

[4] Joyce SA, MacSharry J, Casey PG, Kinsella M, Murphy EF, Shanahan F, Hill C, Gahan CGM 2014 Regulation of host weight gain and lipid metabolism by bacterial bile acid modification in the gut. Proc Natl Acad Sci U S A 111:7421–7426. doi:10.1073/pnas.1323599111. 24799697 PMC4034235

[5] Din MO, Danino T, Prindle A, Skalak M, Selimkhanov J, Allen K, Julio E, Atolia E, Tsimring LS, Bhatia SN, Hasty J 2016 Synchronized cycles of bacterial lysis for in vivo delivery. Nature 536:81–85. doi:10.1038/nature18930. 27437587 PMC5048415

[6] Roy S, Trinchieri G 2017 Microbiota: a key orchestrator of cancer therapy. Nat Rev Cancer 17:271–285. doi:10.1038/nrc.2017.13. 28303904

[7] Peralta-Yahya PP, Zhang FZ, del Cardayre SB, Keasling JD 2012 Microbial engineering for the production of advanced biofuels. Nature 488:320–328. doi:10.1038/nature11478. 22895337

[8] Rodriguez-Brenes IA, Wodarz D, Komarova NL 2013 Stem cell control, oscillations, and tissue regeneration in spatial and non-spatial models. Front Oncol 3:82. doi:10.3389/fonc.2013.00082. 23596567 PMC3625858

[9] Royo JL, Becker PD, Camacho EM, Cebolla A, Link C, Santero E, Guzman CA 2007 In vivo gene regulation in Salmonella spp. by a salicylate-dependent control circuit. Nat Methods 4:937–942. doi:10.1038/nmeth1107. 17922017

[10] Archer EJ, Robinson AB, Suel GM 2012 Engineered E. coli that detect and respond to gut inflammation through nitric oxide sensing. ACS Synth Biol 1:451–457. doi:10.1021/sb3000595. 23656184

[11] Daeffler KNM, Galley JD, Sheth RU, Ortiz-Velez LC, Bibb CO, Shroyer NF, Britton RA, Tabor JJ 2017 Engineering bacterial thiosulfate and tetrathionate sensors for detecting gut inflammation. Mol Syst Biol 13:923. doi:10.15252/msb.20167416. 28373240 PMC5408782

[12] Riglar DT, Giessen TW, Baym M, Kerns SJ, Niederhuber MJ, Bronson RT, Kotula JW, Gerber GK, Way JC, Silver PA 2017 Engineered bacteria can function in the mammalian gut long-term as live diagnostics of inflammation. Nat Biotechnol 35:653. doi:10.1038/nbt.3879. 28553941 PMC5658125

[13] Weissmann G 1991 Aspirin. Sci Am 264:84–90. 10.1038/scientificamerican0191-84 1899486

[14] Winek CL 1994 Winek’s toxicological annual. Allegheny County Department Laboratories, Pittsburgh, PA.

[15] Dalen JE 2006 Aspirin to prevent heart attack and stroke: what's the right dose? Am J Med 119:198–202. doi:10.1016/j.amjmed.2005.11.013. 16490462

[16] Jones RM, Pagmantidis V, Williams PA 2000 sal genes determining the catabolism of salicylate esters are part of a supraoperonic cluster of catabolic genes in Acinetobacter sp. strain ADP1. J Bacteriol 182:2018–2025. doi:10.1128/JB.182.7.2018-2025.2000. 10715011 PMC101918

[17] Zhang D, Zhao Y, He Y, Wang Y, Zhao Y, Zheng Y, Wei X, Zhang L, Li Y, Jin T, Wu L, Wang H, Davison PA, Xu J, Huang WE 2012 Characterization and modeling of transcriptional cross-regulation in Acinetobacter baylyi ADP1. ACS Synth Biol 1:274–283. doi:10.1021/sb3000244. 23651250

[18] Huang WE, Wang H, Zheng HJ, Huang LF, Singer AC, Thompson I, Whiteley AS 2005 Chromosomally located gene fusions constructed in Acinetobacter sp ADP1 for the detection of salicylate. Environ Microbiol 7:1339–1348. doi:10.1111/j.1462-5822.2005.00821.x. 16104857

[19] Grozdanov L, Raasch C, Schulze E, Sonnenborn U, Gottschalk G, Hacker J, Dobrindt U 2004 Analysis of the genome structure of the nonpathogenic probiotic Escherichia coli strain Nissle 1917. J Bacteriol 186:5432–5441. doi:10.1128/JB.186.16.5432-5441.2004. 15292145 PMC490877

[20] Rampley CPN, Davison PA, Qian P, Preston GM, Hunter CN, Thompson IP, Wu LJ, Huang WE 2017 Development of SimCells as a novel chassis for functional biosensors. Sci Rep 7:7261. doi:10.1038/s41598-017-07391-6. 28775370 PMC5543166

[21] Davis JH, Rubin AJ, Sauer RT 2011 Design, construction and characterization of a set of insulated bacterial promoters. Nucleic Acids Res 39:1131–1141. doi:10.1093/nar/gkq810. 20843779 PMC3035448

[22] Alon U 2007 An introduction to systems biology. Design principles of biological circuits. Chapman & Hall/CRC, London, UK.

[23] Schell MA 1993 Molecular biology of the LysR family of transcriptional regulators. Annu Rev Microbiol 47:597. doi:10.1146/annurev.mi.47.100193.003121. 8257110

[24] Maddocks SE, Oyston PCF 2008 Structure and function of the LysR-type transcriptional regulator (LTTR) family proteins. Microbiology 154:3609–3623. doi:10.1099/mic.0.2008/022772-0. 19047729

[25] Lerche M, Dian C, Round A, Lonneborg R, Brzezinski P, Leonard GA 2016 The solution configurations of inactive and activated DntR have implications for the sliding dimer mechanism of LysR transcription factors. Sci Rep 6:19988. doi:10.1038/srep19988. 26817994 PMC4730206

[26] Iizuka R, Yamagishi-Shirasaki M, Funatsu T 2011 Kinetic study of de novo chromophore maturation of fluorescent proteins. Anal Biochem 414:173–178. doi:10.1016/j.ab.2011.03.036. 21459075

[27] Wang BJ, Barahona M, Buck M 2014 Engineering modular and tunable genetic amplifiers for scaling transcriptional signals in cascaded gene networks. Nucleic Acids Res 42:9484–9492. doi:10.1093/nar/gku593. 25030903 PMC4132719

[28] Rogers JK, Guzman CD, Taylor ND, Raman S, Anderson K, Church GM 2015 Synthetic biosensors for precise gene control and real-time monitoring of metabolites. Nucleic Acids Res 43:7648–7660. doi:10.1093/nar/gkv616. 26152303 PMC4551912

[29] Nistala GJ, Wu K, Rao CV, Bhalerao KD 2010 A modular positive feedback-based gene amplifier. J Biol Eng 4:4. doi:10.1186/1754-1611-4-4. 20187959 PMC2845093

[30] Mitrophanov AY, Hadley TJ, Groisman EA 2010 Positive autoregulation shapes response timing and intensity in two-component signal transduction systems. J Mol Biol 401:671–680. doi:10.1016/j.jmb.2010.06.051. 20600106 PMC2942797

[31] Bhalerao KD 2009 Synthetic gene networks: the next wave in biotechnology? Trends Biotechnol 27:368–374. doi:10.1016/j.tibtech.2009.03.003. 19409633

[32] Cho I, Blaser MJ 2012 Applications of next-generation sequencing. The human microbiome: at the interface of health and disease. Nat Rev Genet 13:260–270. doi:10.1038/nrg3182. 22411464 PMC3418802

[33] Mandel MJ, Silhavy TJ 2005 Starvation for different nutrients in Escherichia coli results in differential modulation of RpoS levels and stability. J Bacteriol 187:434–442. doi:10.1128/JB.187.2.434-442.2005. 15629914 PMC543567

[34] Antonczak AK, Simova Z, Tippmann EM 2009 A critical examination of Escherichia coli esterase activity. J Biol Chem 284:28795–28800. doi:10.1074/jbc.M109.027409. 19666472 PMC2781425

[35] Robert S, Lukasz K 2016 The stability and degradation kinetics of acetylsalicylic acid in different organic solutions revisited—an UHPLC–ESI-QTOF spectrometry study. Curr Iss Phar Medi Sci 29:39–41.

[36] Wolfe AJ 2005 The acetate switch. Microbiol Mol Biol Rev 69:12–50. doi:10.1128/MMBR.69.1.12-50.2005. 15755952 PMC1082793

[37] Davis J, Vaughan DH, Cardosi MF 1995 Detection and quantitative determination of catechol derivatives using an iron(III)-ethylenediamine visible absorbance assay. Anal Proc Anal Com 32:423–426. doi:10.1039/AI9953200423.

[38] Whitaker WR, Shepherd ES, Sonnenburg JL 2017 Tunable expression tools enable single-cell strain distinction in the gut microbiome. Cell 169:538. doi:10.1016/j.cell.2017.03.041. 28431251 PMC5576361

[39] Lim B, Zimmermann M, Barry NA, Goodman AL 2017 Engineered regulatory systems modulate gene expression of human commensals in the gut. Cell 169:547. doi:10.1016/j.cell.2017.03.045. 28431252 PMC5532740

[40] Kruis W, Fric P, Pokrotnieks J, Lukas M, Fixa B, Kascak M, Kamm MA, Weismueller J, Beglinger C, Stolte M, Wolff C, Schulze J 2004 Maintaining remission of ulcerative colitis with the probiotic Escherichia coli Nissle 1917 is as effective as with standard mesalazine. Gut 53:1617–1623. doi:10.1136/gut.2003.037747. 15479682 PMC1774300

[41] Schultz M 2008 Clinical use of E. coli Nissle 1917 in inflammatory bowel disease. Inflamm Bowel Dis 14:1012–1018. doi:10.1002/ibd.20377. 18240278

[42] Sonnenborn U, Schulze J 2009 The non-pathogenic Escherichia coli strain Nissle 1917—features of a versatile probiotic. Micro Ecol Health Dis 21:122–158. doi:10.3109/08910600903444267.

[43] Hennekens CH 2002 Update on aspirin in the treatment and prevention of cardiovascular disease. Am J Manag Care 8:S691–S700. 12512736

[44] Yin MJ, Yamamoto Y, Gaynor RB 1998 The anti-inflammatory agents aspirin and salicylate inhibit the activity of I(kappa)B kinase-beta. Nature 396:77. doi:10.1038/23948. 9817203

[45] Mitrophanov AY, Groisman EA 2008 Positive feedback in cellular control systems. Bioessays News Bioessays 30:542–555. doi:10.1002/bies.20769. PMC2486260 18478531

[46] Rosenfeld N, Elowitz MB, Alon U 2002 Negative autoregulation speeds the response times of transcription networks. J Mol Biol 323:785–793. 12417193 10.1016/s0022-2836(02)00994-4

[47] Del VD, Dy AJ, Qian Y 2016 Control theory meets synthetic biology. J R Soc Interface 13:20160380. doi:10.1098/rsif.2016.0380. 27440256 PMC4971224

[48] Cohen SP, Levy SB, Foulds J, Rosner JL 1993 Salicylate induction of antibiotic resistance in Escherichia coli: activation of the Mar operon and a Mar-independent pathway. J Bacteriol 175:7856–7862. 7504664 10.1128/jb.175.24.7856-7862.1993 PMC206962

