# Supplementary material for: Development of Aspirin-Inducible Biosensors in Escherichia coli and SimCells
Source: Appl Environ Microbiol. 2019 Mar 6;85(6):e02959-18. doi: 10.1128/AEM.02959-18 (PMC6414386; doi:10.1128/AEM.02959-18)
Supplement: Supplemental file 1 [file AEM.02959-18-s0001.pdf]

Supplementary information

Characterisation and development of aspirin inducible biosensors in *E. coli* and SimCells

Short title: Aspirin sensor in EcN

Jack Xiaoyu Chen[a, †], Harrison Steel[a, †], Yin-Hu Wu[a,b, †], Yun Wang[a], Jiabao Xu[a], Cordelia P. N. Rampley[a], Ian P. Thompson[a], Antonis Papachristodoulou[a] and Wei E. Huang[a*]

[a]Department of engineering science, Begbroke science park. Oxfordshire, OX5 1PF, United Kingdom

[b]Environmental Simulation and Pollution Control State Key Joint Laboratory, State Environmental Protection Key Laboratory of Microorganism Application and Risk Control (SMARC), School of Environment, Tsinghua University, Beijing 100084, PR China

[c]Department of Engineering Science, University of Oxford, Parks Road, Oxford, OX1 3PJ, United Kingdom

*Corresponding author:
Wei E. Huang
Department of Engineering Science, University of Oxford, Parks Road, OX1 3PJ, Oxford, United Kingdom.
Tel.: +44 1865 283786
Email: wei.huang@eng.ox.ac.uk

[†]These authors contributed equally to this work as the first authors.

Section 1 - Materials and Methods

Gene design

Psal→gfp→salR

E coli optimised salR sequence>
ATGGACCTGTCTCTGATCCGTATCTTCATCTGCGTTTACGAAAACAAAAACATCTCTAAAGC
GGCGGAAATCCTGAACCTGTCTCAGCCGTCTGTTACCTACAACCTGAACCGTCTGCGTAAAC
ACCTGAACAACCCGCTGTTCGAACGTACCCAGTACGGTGTTGAAGCGACCAAACTGTCTCAC
GAACTGTACCCGGTTTTCAAAGAATCTATCCTGAAAATCGAAATCGCGGTTGACGAAGCGCT
GAACTTCAACCCGCTGACCTCTAACAAAACCTTCCGTATCGGTCTGTCTGACATCGGTGAAA
TCTGCCTGCTGCCGACCCTGATCGAATACCTGCGTGCGCACGCGCCGAAAATCAAAATCGAA
GTTGAAGAAATCAAAATCGACCAGGTTGAAAAATGGCTGATCGAAGGTTTCATCGACGTTGC
GGTTTTCAACTCTACCCACCTGGAATTCAAACACCTGGAATACGAAACCCTGTTCCTGGAAC
GTTACGTTGCGCTGGTTAACATGAACCACCCGCGTATCCGTTCTACCCTGTCTTTCGACGCG
TACCTGAACGAATCTCACGTTGCGATCAAATCTTCTACCGGTCACACCCAGGTTGACCACGT
TCTGAAACTGATGGGTCACCAGCGTAAAATCGCGCTGGAAGTTCCGCACTTCGGTGTTCTGC
AGGGTGTTCTGGACAAAACCGACCTGATGGTTACCCTGCCGTCTCGTGCGGCGCAGCAGTAC
CTGAACCAGTCTCACGTTCGTGTTCTGGAACTGCCGTTCCAGATGTCTGAATTCTACGTTGG
TCTGCACTGGTTCGCGCAGACCAACGAACCGCTGGCGCGTATCTGGCTGATCCAGACCTGCA
AAAAAGTTATCTCTGTTCTGTAA

Psal promoter and binding site
>
CCAGCTGATCAGTTGTAGAATGAAAAAATAAGTGCCTGCCAGAATCAAAAAAGTTCCAGCCA
AAAATAGATATTGCATCAAACCTTGGAATGAGCTCAATTCAATCAGTCGTTCATAAAAACGA
GCATTTGTAAAACTTGCTAACCAACAAGCTAGAAGCCCATTAAATGTATTTATAGATAAACT
TACTTTTTTGAATTGTAGCAATTTAAACATCTCAATATCGTTCAGTTATTTGAGGGGTATAG
CTACATTAAAGAGTATGAAAATTAAGAGAAAATTAGTTTTCGCTTCAATGATTCAATTTAAA
TCTAAATATCAA(Bindingsite)TGTTTTAAGATCATAATGATGGATATGAAAACTTAAA
CCTATACCGGATAGGCAATTAAGAAAGAAATATGATATAATT(promoter)GTTTTAAGAT
ATTGATTTTAATAAATTATTTAAAAAATTAATAACTGCTTTAAAAAACTTTAAATTGGATTT
AATTGAGTTTGCAGACTAAAAATAAAACAATAAATCAAGTTCTCAAAGGAAATGAGTC(sal
A→)

GFP>
ATGCGTAAAGGAGAAGAACTTTTCACTGGAGTTGTCCCAATTCTTGTTGAATTAGATGGTGA
TGTTAATGGGCACAAATTTTCTGTCAGTGGAGAGGGTGAAGGTGATGCAACATACGGAAAAC
TTACCCTTAAATTTATTTGCACTACTGGAAAACTACCTGTTCCATGGCCAACACTTGTCACT
ACTCTGACGTATGGTGTTCAATGCTTTTCCCGTTATCCGGATCATATGAAACGGTATGACTT
TTTCAAGAGTGCCATGCCCGAAGGTTATGTACAGGAACGCACTATATCTTTCAAAGATGACG
GGAACTACAAGACGCGTGCTGAAGTCAAGTTTGAAGGTGATACCCTTGTTAATCGTATCGAG
TTAAAAGGTATTGATTTTAAAGAAGATGGAAACATTCTCGGACACAAACTCGAGTACAACTA
TAACTCACACAATGTATACATCACGGCAGACAAACAAAAGAATGGAATCAAAGCTAACTTCA
AAATTCGCCACAACATTGAAGATGGATCCGTTCAACTAGCAGACCATTATCAACAAAATACT
CCAATTGGCGATGGCCCTGTCCTTTTACCAGACAACCATTACCTGTCGACACAATCTGCCCT
TTTGAAAGATCCCAACGAAAAGCGTGACCACATGGTCCTTCTTGAGTTTGTAACTGCTGCTG
GGATTACACATGGCATGGATGAACTATACAAATAA

Psal→gfp→salR>

CCAGCTGATCAGTTGTAGAATGAAAAAATAAGTGCCTGCCAGAATCAAAAAAGTTCCAGCCA
AAAATAGATATTGCATCAAACCTTGGAATGAGCTCAATTCAATCAGTCGTTCATAAAAACGA
GCATTTGTAAAACTTGCTAACCAACAAGCTAGAAGCCCATTAAATGTATTTATAGATAAACT
TACTTTTTTGAATTGTAGCAATTTAAACATCTCAATATCGTTCAGTTATTTGAGGGGTATAG
CTACATTAAAGAGTATGAAAATTAAGAGAAAATTAGTTTTCGCTTCAATGATTCAATTTAAA
TCTAAATATCAA(Bindingsite)TGTTTTAAGATCATAATGATGGATATGAAAACTTAAA
CCTATACCGGATAGGCAATTAAGAAAGAAATATGATATAATT(promoter)GTTTTAAGAT
ATTGATTTTAATAAATTATTTAAAAAATTAATAACTGCTTTAAAAAACTTTAAATTGGATTT
AATTGAGTTTGCAGACTAAAAATAAAACAATAAATCAAGTTCTCAAAGGAAATGAGTCATGC
GTAAAGGAGAAGAACTTTTCACTGGAGTTGTCCCAATTCTTGTTGAATTAGATGGTGATGTT
AATGGGCACAAATTTTCTGTCAGTGGAGAGGGTGAAGGTGATGCAACATACGGAAAACTTAC
CCTTAAATTTATTTGCACTACTGGAAAACTACCTGTTCCATGGCCAACACTTGTCACTACTC
TGACGTATGGTGTTCAATGCTTTTCCCGTTATCCGGATCATATGAAACGGTATGACTTTTTC
AAGAGTGCCATGCCCGAAGGTTATGTACAGGAACGCACTATATCTTTCAAAGATGACGGGAA
CTACAAGACGCGTGCTGAAGTCAAGTTTGAAGGTGATACCCTTGTTAATCGTATCGAGTTAA
AAGGTATTGATTTTAAAGAAGATGGAAACATTCTCGGACACAAACTCGAGTACAACTATAAC
TCACACAATGTATACATCACGGCAGACAAACAAAAGAATGGAATCAAAGCTAACTTCAAAAT
TCGCCACAACATTGAAGATGGATCCGTTCAACTAGCAGACCATTATCAACAAAATACTCCAA
TTGGCGATGGCCCTGTCCTTTTACCAGACAACCATTACCTGTCGACACAATCTGCCCTTTTG
AAAGATCCCAACGAAAAGCGTGACCACATGGTCCTTCTTGAGTTTGTAACTGCTGCTGGGAT
TACACATGGCATGGATGAACTATACAAATAAACGCTAAGAATTTGGCACAAGAGTGTTTTGA
ACGACTTGTGCCTTTAAAACAATTCTATTTTGAAAGAGTTGAATAAAAGTGTTTATGATAGG
ATTAAATTAAAATCATGGAAGATTCTAAAACATGGACCTGTCTCTGATCCGTATCTTCATCT
GCGTTTACGAAAACAAAAACATCTCTAAAGCGGCGGAAATCCTGAACCTGTCTCAGCCGTCT
GTTACCTACAACCTGAACCGTCTGCGTAAACACCTGAACAACCCGCTGTTCGAACGTACCCA
GTACGGTGTTGAAGCGACCAAACTGTCTCACGAACTGTACCCGGTTTTCAAAGAATCTATCC
TGAAAATCGAAATCGCGGTTGACGAAGCGCTGAACTTCAACCCGCTGACCTCTAACAAAACC
TTCCGTATCGGTCTGTCTGACATCGGTGAAATCTGCCTGCTGCCGACCCTGATCGAATACCT
GCGTGCGCACGCGCCGAAAATCAAAATCGAAGTTGAAGAAATCAAAATCGACCAGGTTGAAA
AATGGCTGATCGAAGGTTTCATCGACGTTGCGGTTTTCAACTCTACCCACCTGGAATTCAAA
CACCTGGAATACGAAACCCTGTTCCTGGAACGTTACGTTGCGCTGGTTAACATGAACCACCC
GCGTATCCGTTCTACCCTGTCTTTCGACGCGTACCTGAACGAATCTCACGTTGCGATCAAAT
CTTCTACCGGTCACACCCAGGTTGACCACGTTCTGAAACTGATGGGTCACCAGCGTAAAATC
GCGCTGGAAGTTCCGCACTTCGGTGTTCTGCAGGGTGTTCTGGACAAAACCGACCTGATGGT
TACCCTGCCGTCTCGTGCGGCGCAGCAGTACCTGAACCAGTCTCACGTTCGTGTTCTGGAAC
TGCCGTTCCAGATGTCTGAATTCTACGTTGGTCTGCACTGGTTCGCGCAGACCAACGAACCG
CTGGCGCGTATCTGGCTGATCCAGACCTGCAAAAAAGTTATCTCTGTTCTGTAA

FASTA Psal→gfp→salR>

CCAGCTGATCAGTTGTAGAATGAAAAAATAAGTGCCTGCCAGAATCAAAAAAGTTCCAGCCA
AAAATAGATATTGCATCAAACCTTGGAATGAGCTCAATTCAATCAGTCGTTCATAAAAACGA
GCATTTGTAAAACTTGCTAACCAACAAGCTAGAAGCCCATTAAATGTATTTATAGATAAACT
TACTTTTTTGAATTGTAGCAATTTAAACATCTCAATATCGTTCAGTTATTTGAGGGGTATAG
CTACATTAAAGAGTATGAAAATTAAGAGAAAATTAGTTTTCGCTTCAATGATTCAATTTAAA
TCTAAATATCAATGTTTTAAGATCATAATGATGGATATGAAAACTTAAACCTATACCGGATA
GGCAATTAAGAAAGAAATATGATATAATTGTTTTAAGATATTGATTTTAATAAATTATTTAA
AAAATTAATAACTGCTTTAAAAAACTTTAAATTGGATTTAATTGAGTTTGCAGACTAAAAAT
AAAACAATAAATCAAGTTCTCAAAGGAAATGAGTCATGCGTAAAGGAGAAGAACTTTTCACT
GGAGTTGTCCCAATTCTTGTTGAATTAGATGGTGATGTTAATGGGCACAAATTTTCTGTCAG
TGGAGAGGGTGAAGGTGATGCAACATACGGAAAACTTACCCTTAAATTTATTTGCACTACTG

GAAAACTACCTGTTCCATGGCCAACACTTGTCACTACTCTGACGTATGGTGTTCAATGCTTT
TCCCGTTATCCGGATCATATGAAACGGTATGACTTTTTCAAGAGTGCCATGCCCGAAGGTTA
TGTACAGGAACGCACTATATCTTTCAAAGATGACGGGAACTACAAGACGCGTGCTGAAGTCA
AGTTTGAAGGTGATACCCTTGTTAATCGTATCGAGTTAAAAGGTATTGATTTTAAAGAAGAT
GGAAACATTCTCGGACACAAACTCGAGTACAACTATAACTCACACAATGTATACATCACGGC
AGACAAACAAAAGAATGGAATCAAAGCTAACTTCAAAATTCGCCACAACATTGAAGATGGAT
CCGTTCAACTAGCAGACCATTATCAACAAAATACTCCAATTGGCGATGGCCCTGTCCTTTTA
CCAGACAACCATTACCTGTCGACACAATCTGCCCTTTTGAAAGATCCCAACGAAAAGCGTGA
CCACATGGTCCTTCTTGAGTTTGTAACTGCTGCTGGGATTACACATGGCATGGATGAACTAT
ACAAATAAACGCTAAGAATTTGGCACAAGAGTGTTTTGAACGACTTGTGCCTTTAAAACAAT
TCTATTTTGAAAGAGTTGAATAAAAGTGTTTATGATAGGATTAAATTAAAATCATGGAAGAT
TCTAAAACATGGACCTGTCTCTGATCCGTATCTTCATCTGCGTTTACGAAAACAAAAACATC
TCTAAAGCGGCGGAAATCCTGAACCTGTCTCAGCCGTCTGTTACCTACAACCTGAACCGTCT
GCGTAAACACCTGAACAACCCGCTGTTCGAACGTACCCAGTACGGTGTTGAAGCGACCAAAC
TGTCTCACGAACTGTACCCGGTTTTCAAAGAATCTATCCTGAAAATCGAAATCGCGGTTGAC
GAAGCGCTGAACTTCAACCCGCTGACCTCTAACAAAACCTTCCGTATCGGTCTGTCTGACAT
CGGTGAAATCTGCCTGCTGCCGACCCTGATCGAATACCTGCGTGCGCACGCGCCGAAAATCA
AAATCGAAGTTGAAGAAATCAAAATCGACCAGGTTGAAAAATGGCTGATCGAAGGTTTCATC
GACGTTGCGGTTTTCAACTCTACCCACCTGGAATTCAAACACCTGGAATACGAAACCCTGTT
CCTGGAACGTTACGTTGCGCTGGTTAACATGAACCACCCGCGTATCCGTTCTACCCTGTCTT
TCGACGCGTACCTGAACGAATCTCACGTTGCGATCAAATCTTCTACCGGTCACACCCAGGTT
GACCACGTTCTGAAACTGATGGGTCACCAGCGTAAAATCGCGCTGGAAGTTCCGCACTTCGG
TGTTCTGCAGGGTGTTCTGGACAAAACCGACCTGATGGTTACCCTGCCGTCTCGTGCGGCGC
AGCAGTACCTGAACCAGTCTCACGTTCGTGTTCTGGAACTGCCGTTCCAGATGTCTGAATTC
TACGTTGGTCTGCACTGGTTCGCGCAGACCAACGAACCGCTGGCGCGTATCTGGCTGATCCA
GACCTGCAAAAAAGTTATCTCTGTTCTGTAA

Designed Psal→gfp→salR>
CTTCAAAGATCTAATTTAAATCTAAATATCAATGTTTTAAGATCATAATGATGGATATGAAA
ACTTAAACCTATACCGGATAGGCAATTAAGAAAGAAATATGATATAATTGTTTTAAGATATT
GATTTTAATAAATTATTTAAAAAATTAATAACTGCTTTAAAAAACTTTAAATTGGATTTAAT
TGAGTTTGCAGACTAAAAATAAAACAATAAATCAAGCTTCTCAAAGGAAATGAGTCATGCGT
AAAGGAGAAGAACTTTTCACTGGAGTTGTCCCAATTCTTGTTGAATTAGATGGTGATGTTAA
TGGGCACAAATTTTCTGTCAGTGGAGAGGGTGAAGGTGATGCAACATACGGAAAACTTACCC
TTAAATTTATTTGCACTACTGGAAAACTACCTGTTCCATGGCCAACACTTGTCACTACTCTG
ACGTATGGTGTTCAATGCTTTTCCCGTTATCCGGATCATATGAAACGGTATGACTTTTTCAA
GAGTGCCATGCCCGAAGGTTATGTACAGGAACGCACTATATCTTTCAAAGATGACGGGAACT
ACAAGACGCGTGCTGAAGTCAAGTTTGAAGGTGATACCCTTGTTAATCGTATCGAGTTAAAA
GGTATTGATTTTAAAGAAGATGGAAACATTCTCGGACACAAACTCGAGTACAACTATAACTC
ACACAATGTATACATCACGGCAGACAAACAAAAGAATGGAATCAAAGCTAACTTCAAAATTC
GCCACAACATTGAAGATGGATCCGTTCAACTAGCAGACCATTATCAACAAAATACTCCAATT
GGCGATGGCCCTGTCCTTTTACCAGACAACCATTACCTGTCGACACAATCTGCCCTTTTGAA
AGATCCCAACGAAAAGCGTGACCACATGGTCCTTCTTGAGTTTGTAACTGCTGCTGGGATTA
CACATGGCATGGATGAACTATACAAATAAACGCTAAGCTTTGGCACAAGAGTGTTTTGAACG
ACTTGTGCCTTTAAAACAATTCTATTTTGAAAGAGTTGAATAAAAGTGTTTATGATAGGATT
AAATTAAAATCATGGAAGATTCTAAAACATGGACCTGTCTCTGATCCGTATCTTCATCTGCG
TTTACGAAAACAAAAACATCTCTAAAGCGGCGGAAATCCTGAACCTGTCTCAGCCGTCTGTT
ACCTACAACCTGAACCGTCTGCGTAAACACCTGAACAACCCGCTGTTCGAACGTACCCAGTA
CGGTGTTGAAGCGACCAAACTGTCTCACGAACTGTACCCGGTTTTCAAAGAATCTATCCTGA
AAATCGAAATCGCGGTTGACGAAGCGCTGAACTTCAACCCGCTGACCTCTAACAAAACCTTC

CGTATCGGTCTGTCTGACATCGGTGAAATCTGCCTGCTGCCGACCCTGATCGAATACCTGCG
TGCGCACGCGCCGAAAATCAAAATCGAAGTTGAAGAAATCAAAATCGACCAGGTTGAAAAAT
GGCTGATCGAAGGTTTCATCGACGTTGCGGTTTTCAACTCTACCCACCTGGAATTCAAACAC
CTGGAATACGAAACCCTGTTCCTGGAACGTTACGTTGCGCTGGTTAACATGAACCACCCGCG
TATCCGTTCTACCCTGTCTTTCGACGCGTACCTGAACGAATCTCACGTTGCGATCAAATCTT
CTACCGGTCACACCCAGGTTGACCACGTTCTGAAACTGATGGGTCACCAGCGTAAAATCGCG
CTGGAAGTTCCGCACTTCGGTGTTCTGCAGGGTGTTCTGGACAAAACCGACCTGATGGTTAC
CCTGCCGTCTCGTGCGGCGCAGCAGTACCTGAACCAGTCTCACGTTCGTGTTCTGGAACTGC
CGTTCCAGATGTCTGAATTCTACGTTGGTCTGCACTGGTTCGCGCAGACCAACGAACCGCTG
GCGCGTATCTGGCTGATCCAGACCTGCAAAAAAGTTATCTCTGTTCTGTAAAGATCTAAACA
ATTAA

salR←Psal→gfp

E coli optimised salR sequence>
ATGGACCTGTCTCTGATCCGTATCTTCATCTGCGTTTACGAAAACAAAAACATCTCTAAAGC
GGCGGAAATCCTGAACCTGTCTCAGCCGTCTGTTACCTACAACCTGAACCGTCTGCGTAAAC
ACCTGAACAACCCGCTGTTCGAACGTACCCAGTACGGTGTTGAAGCGACCAAACTGTCTCAC
GAACTGTACCCGGTTTTCAAAGAATCTATCCTGAAAATCGAAATCGCGGTTGACGAAGCGCT
GAACTTCAACCCGCTGACCTCTAACAAAACCTTCCGTATCGGTCTGTCTGACATCGGTGAAA
TCTGCCTGCTGCCGACCCTGATCGAATACCTGCGTGCGCACGCGCCGAAAATCAAAATCGAA
GTTGAAGAAATCAAAATCGACCAGGTTGAAAAATGGCTGATCGAAGGTTTCATCGACGTTGC
GGTTTTCAACTCTACCCACCTGGAATTCAAACACCTGGAATACGAAACCCTGTTCCTGGAAC
GTTACGTTGCGCTGGTTAACATGAACCACCCGCGTATCCGTTCTACCCTGTCTTTCGACGCG
TACCTGAACGAATCTCACGTTGCGATCAAATCTTCTACCGGTCACACCCAGGTTGACCACGT
TCTGAAACTGATGGGTCACCAGCGTAAAATCGCGCTGGAAGTTCCGCACTTCGGTGTTCTGC
AGGGTGTTCTGGACAAAACCGACCTGATGGTTACCCTGCCGTCTCGTGCGGCGCAGCAGTAC
CTGAACCAGTCTCACGTTCGTGTTCTGGAACTGCCGTTCCAGATGTCTGAATTCTACGTTGG
TCTGCACTGGTTCGCGCAGACCAACGAACCGCTGGCGCGTATCTGGCTGATCCAGACCTGCA
AAAAAGTTATCTCTGTTCTGTAA

Constitutive promoter, proD (insulated promoter)
>ttctagagCACAGCTAACACCACGTCGTCCCTATCTGCTGCCCTAGGTCTATGAGTGGTTG
CTGGATAACTTTACGGGCATGCATAAGGCTCGTATAATATATTCAGGGAGACCACAACGGTT
TCCCTCTACAAATAATTTTGTTTAACTTTtactagag

Constitutive promoter structure
Plasmid constructs contained the following elements:
promoter-TACTAGAG-B0032-TACTAG-ORF(dsRed,GFP, Gemini)-
TACTAGAG-B0015, where the ORF was exchanged using standard
PCR-based techniques.

B0032>>BBa_B0032 Part-only sequence (13 bp) ribosome binding
site>
tcacacaggaaag

B0015>>BBa_B0015 Part-only sequence (129 bp) Terminator
ccaggcatcaaataaaacgaaaggctcagtcgaaagactgggcctttcgttttatctgttgt
ttgtcggtgaacgctctctactagagtcacactggctcaccttcgggtgggcctttctgcgt
ttata

Constitutive promoter
>ttctagagCACAGCTAACACCACGTCGTCCCTATCTGCTGCCCTAGGTCTATGAGTGGTTG
CTGGATAACTTTACGGGCATGCATAAGGCTCGTATAATATATTCAGGGAGACCACAACGGTT
TCCCTCTACAAATAATTTTGTTTAACTTTtactagagtcacacaggaaagTACTAG(atg)

Psal promoter and binding site
>
CCAGCTGATCAGTTGTAGAATGAAAAAATAAGTGCCTGCCAGAATCAAAAAAGTTCCAGCCA
AAAATAGATATTGCATCAAACCTTGGAATGAGCTCAATTCAATCAGTCGTTCATAAAAACGA
GCATTTGTAAAACTTGCTAACCAACAAGCTAGAAGCCCATTAAATGTATTTATAGATAAACT
TACTTTTTTGAATTGTAGCAATTTAAACATCTCAATATCGTTCAGTTATTTGAGGGGTATAG

CTACATTAAAGAGTATGAAAATTAAGAGAAAATTAGTTTTCGCTTCAATGATTCAATTTAAA
TCTAAATATCAA(Bindingsite)TGTTTTAAGATCATAATGATGGATATGAAAACTTAAA
CCTATACCGGATAGGCAATTAAGAAAGAAATATGATATAATT(promoter)GTTTTAAGAT
ATTGATTTTAATAAATTATTTAAAAAATTAATAACTGCTTTAAAAAACTTTAAATTGGATTT
AATTGAGTTTGCAGACTAAAAATAAAACAATAAATCAAGTTCTCAAAGGAAATGAGTC(sal
A→)

GFP>
ATGCGTAAAGGAGAAGAACTTTTCACTGGAGTTGTCCCAATTCTTGTTGAATTAGATGGTGA
TGTTAATGGGCACAAATTTTCTGTCAGTGGAGAGGGTGAAGGTGATGCAACATACGGAAAAC
TTACCCTTAAATTTATTTGCACTACTGGAAAACTACCTGTTCCATGGCCAACACTTGTCACT
ACTCTGACGTATGGTGTTCAATGCTTTTCCCGTTATCCGGATCATATGAAACGGTATGACTT
TTTCAAGAGTGCCATGCCCGAAGGTTATGTACAGGAACGCACTATATCTTTCAAAGATGACG
GGAACTACAAGACGCGTGCTGAAGTCAAGTTTGAAGGTGATACCCTTGTTAATCGTATCGAG
TTAAAAGGTATTGATTTTAAAGAAGATGGAAACATTCTCGGACACAAACTCGAGTACAACTA
TAACTCACACAATGTATACATCACGGCAGACAAACAAAAGAATGGAATCAAAGCTAACTTCA
AAATTCGCCACAACATTGAAGATGGATCCGTTCAACTAGCAGACCATTATCAACAAAATACT
CCAATTGGCGATGGCCCTGTCCTTTTACCAGACAACCATTACCTGTCGACACAATCTGCCCT
TTTGAAAGATCCCAACGAAAAGCGTGACCACATGGTCCTTCTTGAGTTTGTAACTGCTGCTG
GGATTACACATGGCATGGATGAACTATACAAATAA

Pcon→SalR>
>ttctagagCACAGCTAACACCACGTCGTCCCTATCTGCTGCCCTAGGTCTATGAGTGGTTG
CTGGATAACTTTACGGGCATGCATAAGGCTCGTATAATATATTCAGGGAGACCACAACGGTT
TCCCTCTACAAATAATTTTGTTTAACTTTtactagagtcacacaggaaagTACTAGATGGAC
CTGTCTCTGATCCGTATCTTCATCTGCGTTTACGAAAACAAAAACATCTCTAAAGCGGCGGA
AATCCTGAACCTGTCTCAGCCGTCTGTTACCTACAACCTGAACCGTCTGCGTAAACACCTGA
ACAACCCGCTGTTCGAACGTACCCAGTACGGTGTTGAAGCGACCAAACTGTCTCACGAACTG
TACCCGGTTTTCAAAGAATCTATCCTGAAAATCGAAATCGCGGTTGACGAAGCGCTGAACTT
CAACCCGCTGACCTCTAACAAAACCTTCCGTATCGGTCTGTCTGACATCGGTGAAATCTGCC
TGCTGCCGACCCTGATCGAATACCTGCGTGCGCACGCGCCGAAAATCAAAATCGAAGTTGAA
GAAATCAAAATCGACCAGGTTGAAAAATGGCTGATCGAAGGTTTCATCGACGTTGCGGTTTT
CAACTCTACCCACCTGGAATTCAAACACCTGGAATACGAAACCCTGTTCCTGGAACGTTACG
TTGCGCTGGTTAACATGAACCACCCGCGTATCCGTTCTACCCTGTCTTTCGACGCGTACCTG
AACGAATCTCACGTTGCGATCAAATCTTCTACCGGTCACACCCAGGTTGACCACGTTCTGAA
ACTGATGGGTCACCAGCGTAAAATCGCGCTGGAAGTTCCGCACTTCGGTGTTCTGCAGGGTG
TTCTGGACAAAACCGACCTGATGGTTACCCTGCCGTCTCGTGCGGCGCAGCAGTACCTGAAC
CAGTCTCACGTTCGTGTTCTGGAACTGCCGTTCCAGATGTCTGAATTCTACGTTGGTCTGCA
CTGGTTCGCGCAGACCAACGAACCGCTGGCGCGTATCTGGCTGATCCAGACCTGCAAAAAAG
TTATCTCTGTTCTGTAA

Reverse complement Pcon→salR>
>Pcon-SalR reverse complement
TTACAGAACAGAGATAACTTTTTTGCAGGTCTGGATCAGCCAGATACGCGCCAGCGGTTCGT
TGGTCTGCGCGAACCAGTGCAGACCAACGTAGAATTCAGACATCTGGAACGGCAGTTCCAGA
ACACGAACGTGAGACTGGTTCAGGTACTGCTGCGCCGCACGAGACGGCAGGGTAACCATCAG
GTCGGTTTTGTCCAGAACACCCTGCAGAACACCGAAGTGCGGAACTTCCAGCGCGATTTTAC
GCTGGTGACCCATCAGTTTCAGAACGTGGTCAACCTGGGTGTGACCGGTAGAAGATTTGATC
GCAACGTGAGATTCGTTCAGGTACGCGTCGAAAGACAGGGTAGAACGGATACGCGGGTGGTT
CATGTTAACCAGCGCAACGTAACGTTCCAGGAACAGGGTTTCGTATTCCAGGTGTTTGAATT
CCAGGTGGGTAGAGTTGAAAACCGCAACGTCGATGAAACCTTCGATCAGCCATTTTTCAACC

TGGTCGATTTTGATTTCTTCAACTTCGATTTTGATTTTCGGCGCGTGCGCACGCAGGTATTC
GATCAGGGTCGGCAGCAGGCAGATTTCACCGATGTCAGACAGACCGATACGGAAGGTTTTGT
TAGAGGTCAGCGGGTTGAAGTTCAGCGCTTCGTCAACCGCGATTTCGATTTTCAGGATAGAT
TCTTTGAAAACCGGGTACAGTTCGTGAGACAGTTTGGTCGCTTCAACACCGTACTGGGTACG
TTCGAACAGCGGGTTGTTCAGGTGTTTACGCAGACGGTTCAGGTTGTAGGTAACAGACGGCT
GAGACAGGTTCAGGATTTCCGCCGCTTTAGAGATGTTTTTGTTTTCGTAAACGCAGATGAAG
ATACGGATCAGAGACAGGTCCATCTAGTActttcctgtgtgactctagtaAAAGTTAAACAA
AATTATTTGTAGAGGGAAACCGTTGTGGTCTCCCTGAATATATTATACGAGCCTTATGCATG
CCCGTAAAGTTATCCAGCAACCACTCATAGACCTAGGGCAGCAGATAGGGACGACGTGGTGT
TAGCTGTGctctagaa

Designed salR←Psal→gfp
TAAAGATCTAAACAATTAATTACAGAACAGAGATAACTTTTTTGCAGGTCTGGATCAGCCAG
ATACGCGCCAGCGGTTCGTTGGTCTGCGCGAACCAGTGCAGACCAACGTAGAATTCAGACAT
CTGGAACGGCAGTTCCAGAACACGAACGTGAGACTGGTTCAGGTACTGCTGCGCCGCACGAG
ACGGCAGGGTAACCATCAGGTCGGTTTTGTCCAGAACACCCTGCAGAACACCGAAGTGCGGA
ACTTCCAGCGCGATTTTACGCTGGTGACCCATCAGTTTCAGAACGTGGTCAACCTGGGTGTG
ACCGGTAGAAGATTTGATCGCAACGTGAGATTCGTTCAGGTACGCGTCGAAAGACAGGGTAG
AACGGATACGCGGGTGGTTCATGTTAACCAGCGCAACGTAACGTTCCAGGAACAGGGTTTCG
TATTCCAGGTGTTTGAATTCCAGGTGGGTAGAGTTGAAAACCGCAACGTCGATGAAACCTTC
GATCAGCCATTTTTCAACCTGGTCGATTTTGATTTCTTCAACTTCGATTTTGATTTTCGGCG
CGTGCGCACGCAGGTATTCGATCAGGGTCGGCAGCAGGCAGATTTCACCGATGTCAGACAGA
CCGATACGGAAGGTTTTGTTAGAGGTCAGCGGGTTGAAGTTCAGCGCTTCGTCAACCGCGAT
TTCGATTTTCAGGATAGATTCTTTGAAAACCGGGTACAGTTCGTGAGACAGTTTGGTCGCTT
CAACACCGTACTGGGTACGTTCGAACAGCGGGTTGTTCAGGTGTTTACGCAGACGGTTCAGG
TTGTAGGTAACAGACGGCTGAGACAGGTTCAGGATTTCCGCCGCTTTAGAGATGTTTTTGTT
TTCGTAAACGCAGATGAAGATACGGATCAGAGACAGGTCCATCTAGTActttcctgtgtgac
tctagtaAAAGTTAAACAAAATTATTTGTAGAGGGAAACCGTTGTGGTCTCCCTGAATATAT
TATACGAGCCTTATGCATGCCCGTAAAGTTATCCAGCAACCACTCATAGACCTAGGGCAGCA
GATAGGGACGACGTGGTGTTAGCTGTGctctagaaCTTCAAAATTTAAATCTAAATATCAAT
GTTTTAAGATCATAATGATGGATATGAAAACTTAAACCTATACCGGATAGGCAATTAAGAAA
GAAATATGATATAATTGTTTTAAGATATTGATTTTAATAAATTATTTAAAAAATTAATAACT
GCTTTAAAAAACTTTAAATTGGATTTAATTGAGTTTGCAGACTAAAAATAAAACAATAAATC
AAGCTTCTCAAAGGAAATGAGTCATGCGTAAAGGAGAAGAACTTTTCACTGGAGTTGTCCCA
ATTCTTGTTGAATTAGATGGTGATGTTAATGGGCACAAATTTTCTGTCAGTGGAGAGGGTGA
AGGTGATGCAACATACGGAAAACTTACCCTTAAATTTATTTGCACTACTGGAAAACTACCTG
TTCCATGGCCAACACTTGTCACTACTCTGACGTATGGTGTTCAATGCTTTTCCCGTTATCCG
GATCATATGAAACGGTATGACTTTTTCAAGAGTGCCATGCCCGAAGGTTATGTACAGGAACG
CACTATATCTTTCAAAGATGACGGGAACTACAAGACGCGTGCTGAAGTCAAGTTTGAAGGTG
ATACCCTTGTTAATCGTATCGAGTTAAAAGGTATTGATTTTAAAGAAGATGGAAACATTCTC
GGACACAAACTCGAGTACAACTATAACTCACACAATGTATACATCACGGCAGACAAACAAAA
GAATGGAATCAAAGCTAACTTCAAAATTCGCCACAACATTGAAGATGGATCCGTTCAACTAG
CAGACCATTATCAACAAAATACTCCAATTGGCGATGGCCCTGTCCTTTTACCAGACAACCAT
TACCTGTCGACACAATCTGCCCTTTTGAAAGATCCCAACGAAAAGCGTGACCACATGGTCCT
TCTTGAGTTTGTAACTGCTGCTGGGATTACACATGGCATGGATGAACTATACAAATAAACGC
TAAGCTTAGATCTTGG

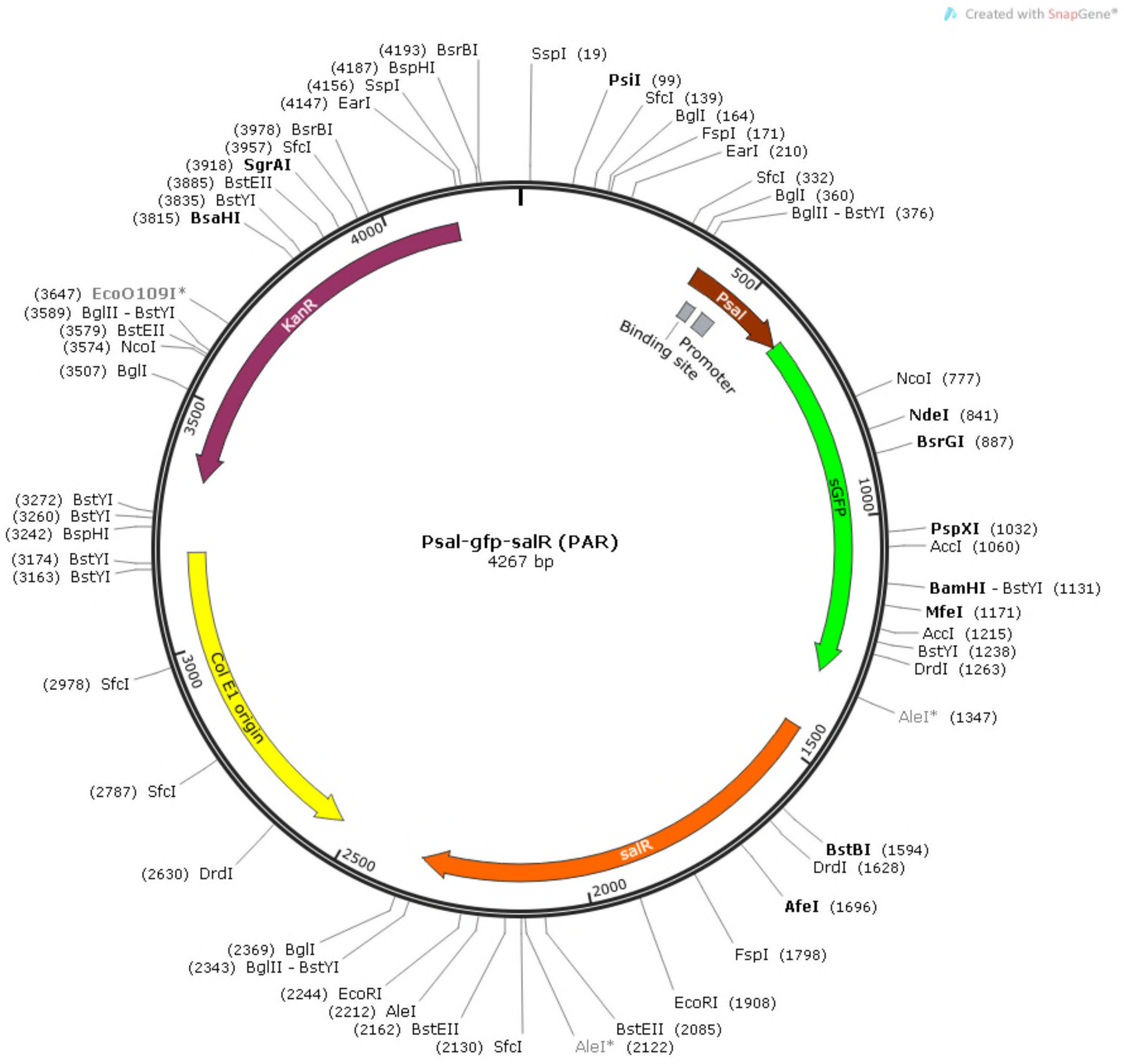

Plasmid map of Psal-gfp-salR (PAR)

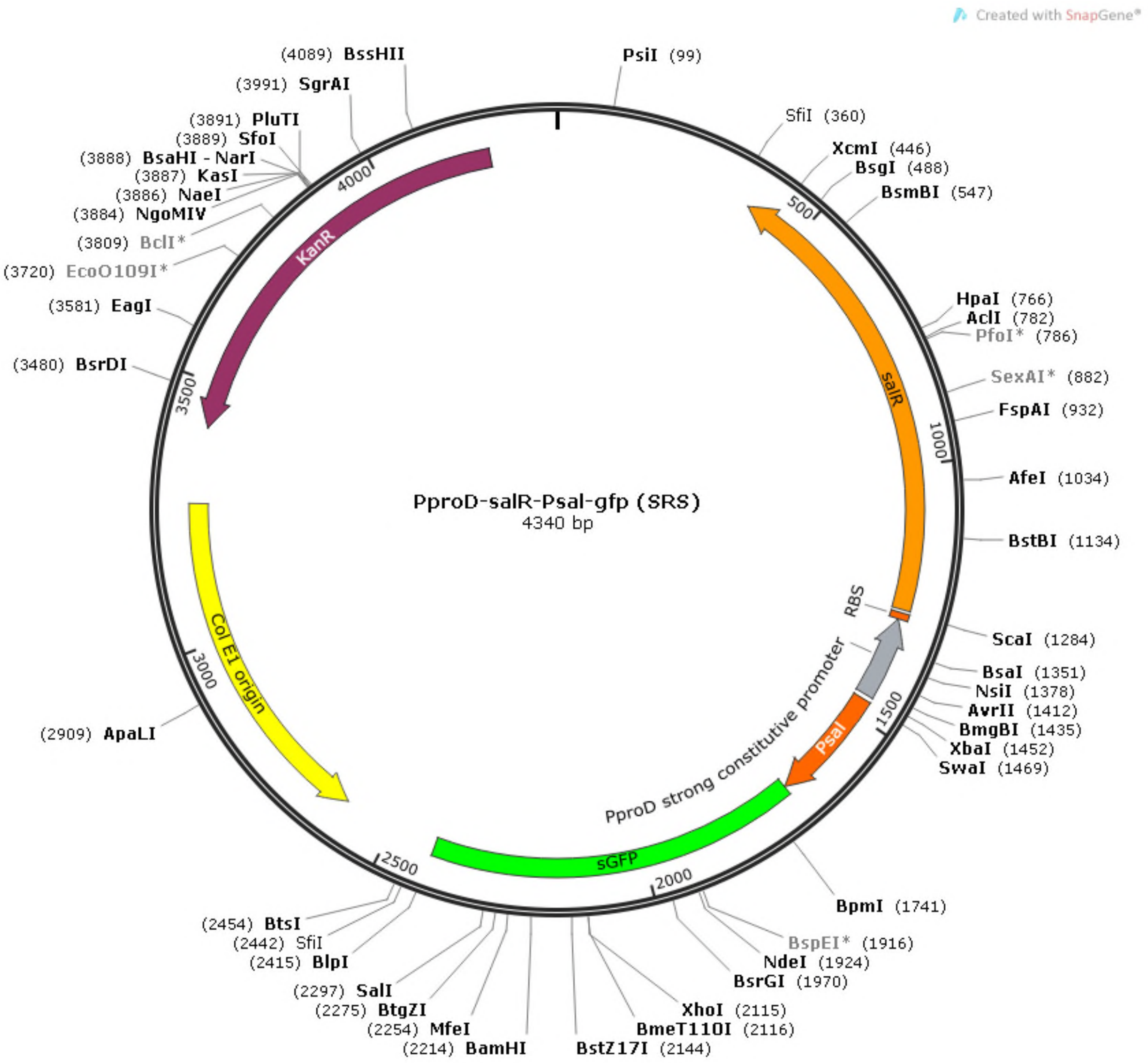

Plasmid map of PproD-salR-Psal-gfp (SRS)

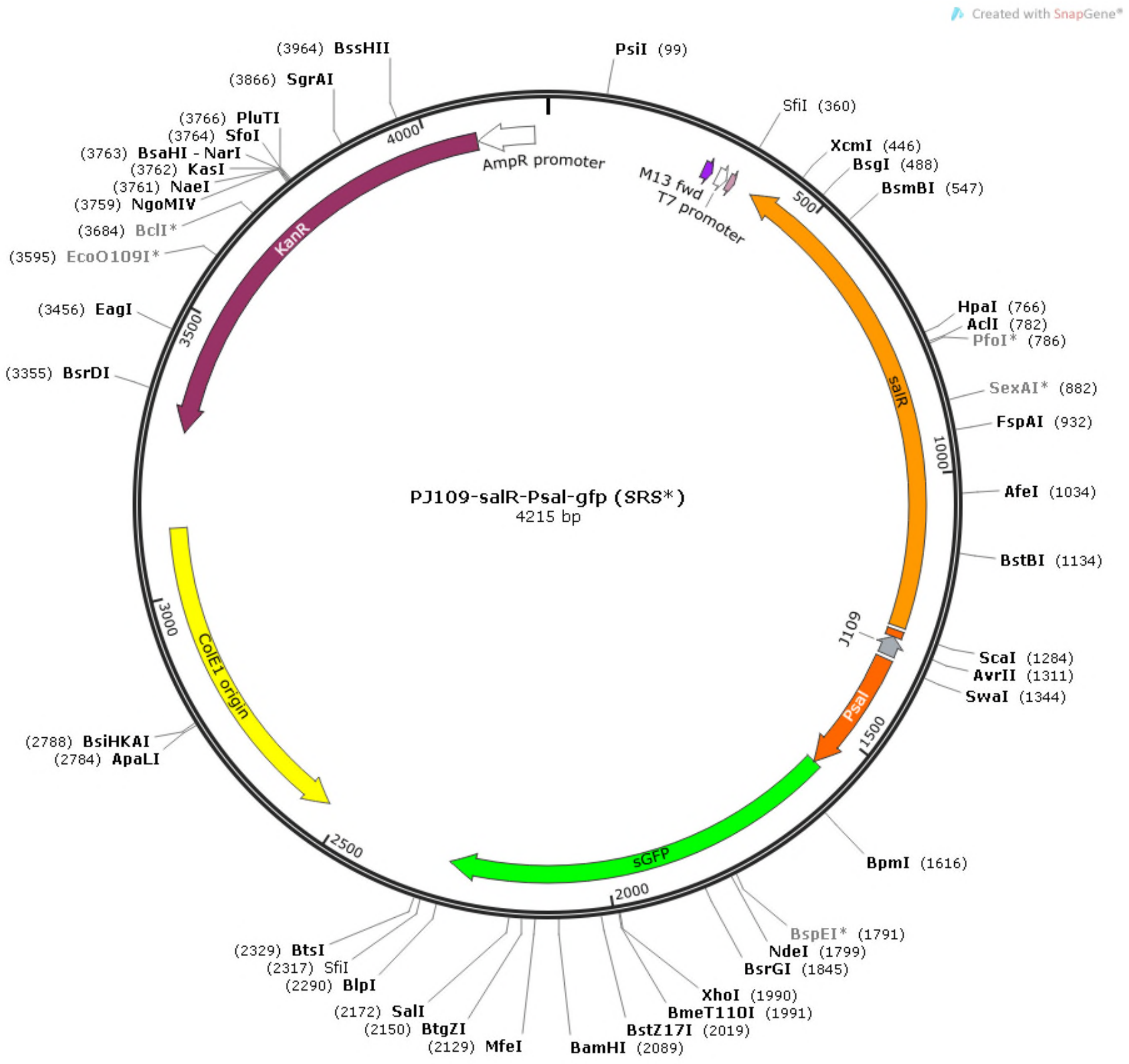

Plasmid map of PJ109-salR-Psal-gfp (SRS*)

Section 2 - Mathematical Modelling

We developed a mathematical model of our system following traditional modelling approaches for gene circuits[1], aiming to simulate its behaviour so that we can examine the hypothesised interactions within our system. Ideally our models would be able to capture the differences in behaviour between the PAR, SRS, SRS* circuits as level of induction changes. Our model (see discussion in main text) reflects the architectures outlined in Figure 2, and uses least-squares parameter fitting to determine parameter values. We observed that the fit model was able to accurately capture both the dose- and dynamic-response experimental data, thereby supporting the assumptions upon which the model was designed.

S2.1 Model Structure

Our model describes our system using differential equations that govern the behaviour of two variables, $[salR]$ the concentration of protein regulator *salR*, and $[GFP]$ the concentration of the fluorescent protein which represents the system's measurable output. The concentration of $[salR]$ is governed by different equations depending upon the system architecture:

$$\frac{d[salR]}{dt} = S([a],[salR])\alpha_1 - \delta_1[salR] \qquad \text{PAR System} \qquad (1a)$$

$$\frac{d[salR]}{dt} = \alpha_2 - \delta_1[salR] \qquad \text{SRS System, ProD} \qquad (1b)$$

$$\frac{d[salR]}{dt} = \alpha_2^* - \delta_1[salR] \qquad \text{SRS System, J109} \qquad (1c)$$

where $[a]$ is inducer (i.e. Asprin) concentration. The system output (GFP) is governed (in all systems) by:

$$\frac{d[GFP]}{dt} = S([a],[salR])\alpha_3 - \delta_2[GFP] \qquad \text{All Systems} \qquad (2)$$

The regulating function $S([a],[salR])$ describes the interaction between SalR, inducer concentration, and the Psal promoter, and is given by:

$$S([a],[salR]) = \frac{K_R}{K_R+[salR]}\left(L + (1-L)\frac{[salR]}{[salR]+K_A}\frac{[a]^n}{[a]^n+K_a}\right) \qquad (3)$$

Which includes typical hill function terms[1] to account for the two potential binding modes of salR; repressive (in the absence of a), and activating (in the presence ot a). This equation also accounts for promoter leakage, L, when no salR is present. We can examine the behaviour of (3) by considering various limiting cases: When no salR is present we have $([a],[salR]) \to L$, the unregulated output from the promoter. When salR is very large we have $S([a],[salR]) \to 0$ regardless of inducer concentration, which is supported by our experiments with the SRS system and a strong RBS demonstrating that in the absence of inducer salR strongly represses proD. When salR concentration is moderate (i.e. $K_A < [salR] < K_R$) the system responds to inducer concentration$[a]$, and thus when we have $S([a],[salR]) \to 1$. The other parameters values in equations (1)-(3) are defined in Table S2 below.

Table S2

Parameter	Description	LB Media	Units
δ	Degradation rate of proteins.	3.5×10^{-5}	1/s
α_1	Combined transcription/translation rate of salR in the PAR system.	6.02×10^{-3}	nM/s
α_2	Combined transcription/translation rate of salR in the SRS system.	0.153	nM/s
α_2^*	Combined transcription/translation rate of salR in the SRS* system.	0.0217	nM/s
α_3	Combined transcription/translation rate of GFP in all systems.	1.88	nM/s
K_R	Equilibrium binding constant of salR in its repressive (non-induced) form.	3.75×10^2	nM
K_A	Equilibrium binding constant of salR in its activating (induced) form.	10	nM
K_a	Equilibrium binding constant of binding between Asprin and salR.	0.457	μM
n	Exponent for hill function governing Asprin-salR binding.	1	none
L	Transcriptional leakage rate from Psal as a proportion of maximal transcription output.	0.104	none

S2.2 Simulation Approach

Our model is implemented in MATLAB 2017b; numerical integration is performed a Runge-Kutta 4th method, and parameters are fit using the *fmincon* function. Simulation start-time corresponds to addition of $[a]$. We therefore assume that initially $[GFP]_0 \approx 0$ in all systems. The initial salR concentration, $[salR]_0$, is determined by solving (1a) (with $[a] = 0$) or (1b,c) at steady-state with the parameters fit for the LB growth media. This is because all cells were grown in LB prior to commencement of each experiment.

S2.3 Parameter Fitting

Parameter values (listed in Table S2 along with definitions) were fit to experimental data as follows. Because there is insufficient experimental data to identify all parameters in our model, we set $\boldsymbol{n = 1}$ as which corresponds to non-cooperative binding of the inducer$\boldsymbol{a}$. Exponents of $[\boldsymbol{salR}]$ are likewise unity throughout, though lack of cooperativity in binding Psal does not imply that salR does not dimerise quickly (compared to other binding processes) once it is expressed. The value of$\boldsymbol{K_A}$ was set to an arbitrary value of $\boldsymbol{10\,nM}$, meaning that the fit value of $\boldsymbol{K_R}$ governs the relative strength of repressive vs activating binding of salR, and expression rates of salR in each system $(\boldsymbol{\alpha_1, \alpha_2, \alpha_2^*})$ govern the absolute level of salR-promoter interaction. The free parameters ($\boldsymbol{\delta, \alpha_1, \alpha_2, \alpha_2^*, \alpha_3, K_R, K_a, L}$) were then least-squares fit to the experimental data for each circuit variant (Figures 3,4 of the main text).

The resulting parameters (Table S2) demonstrate some underlying phenomena. We observe that the fit δ value corresponds to a protein half-life of approximately 5.5 hours, in the ballpark expected for relatively stable proteins. The ratio of transcription for the SRS/SRS* circuits is $\alpha_2/\alpha_2^* \approx 7$, which is a similar factor of change to that observed in Figure 4C of the main text. As expected we find that $K_R \gg K_A$, supporting our operational hypothesis that binding of salR in its active (inducer bound) form to Psal is favourable compared to binding of of inactive salR. Furthermore, the equilibrium constant for salR-Inducer binding (K_a) falls into the middle of the range of inducer concentrations tested, where we anticipate salR action is becoming dominated by its active form.

S2.4 Supplementary References

1 - Ingalls, B. P. Mathematical Modelling in Systems Biology : An Introduction. J. Chem. Inf. Model. 53, 1–396 (2014).

Characterisation and development of aspirin inducible biosensors in *E. coli* and SimCells

Supplementary figures

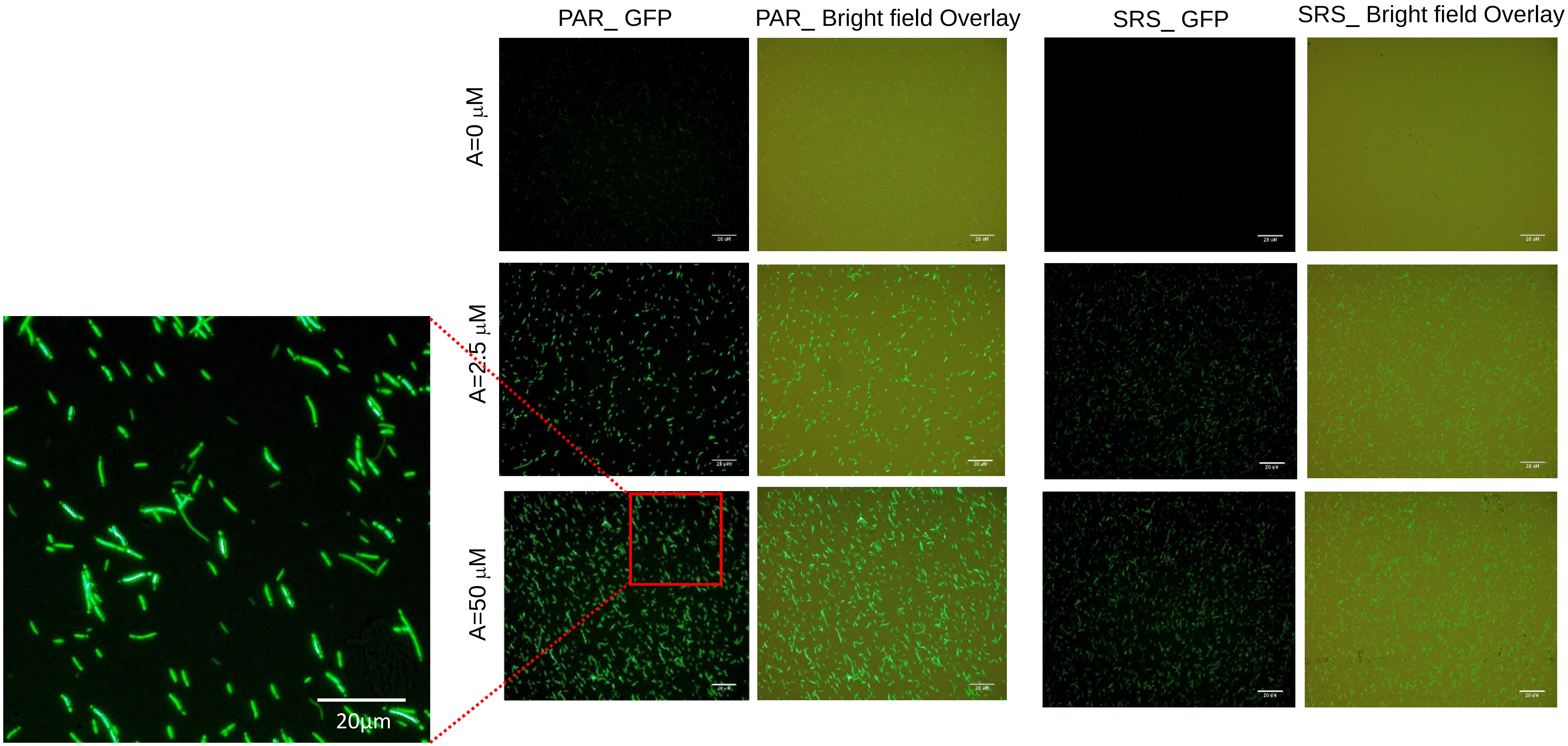

Figure S1. A) Microscope images of GFP expression of SRS and PAR circuits in *E. coli* DH5a. Images were taken at both GFP channel and overlaid with bright field channel for indication of potential mutated circuits or non-transformed bacterial cells. The specific concentration of aspirin is indicated beside the plot. On the left, enlarged images of the PAR circuit when induced with aspirin (50 μM) clearly show two separate populations with different GFP intensity (High/Low) as reflected in result from flow cytometry.

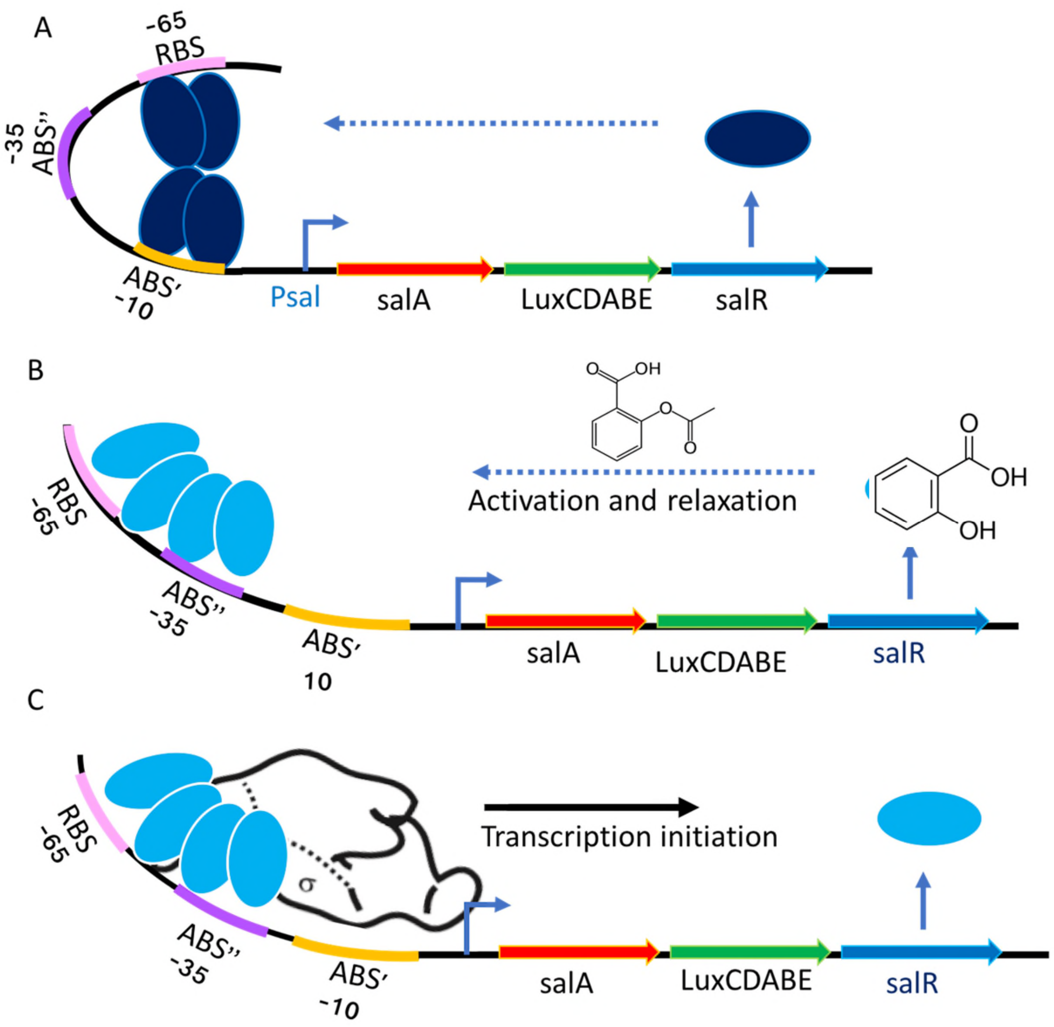

Figure S2. A potential schematic for the salR "sliding dimer" regulation mechanism. (A) In the absence of SA, salR regulates the expression of the P_{sal} operon by binding the promoter region at three different functional subsites: a high affinity Repression binding site (RBS), often found near position -65 relative to the transcription start site and two low affinity activation binding sites (ABS' and ABS'') found near positions - 10 and -35 respectively. (B) upon activation by aspirin binding, a shift in promoter region binding sites from RBS/ABS' to RBS/ABS'' releases the -25 box of the promoter region DNA and leading to (C) RNA polymerase recognition and subsequent gene expression.

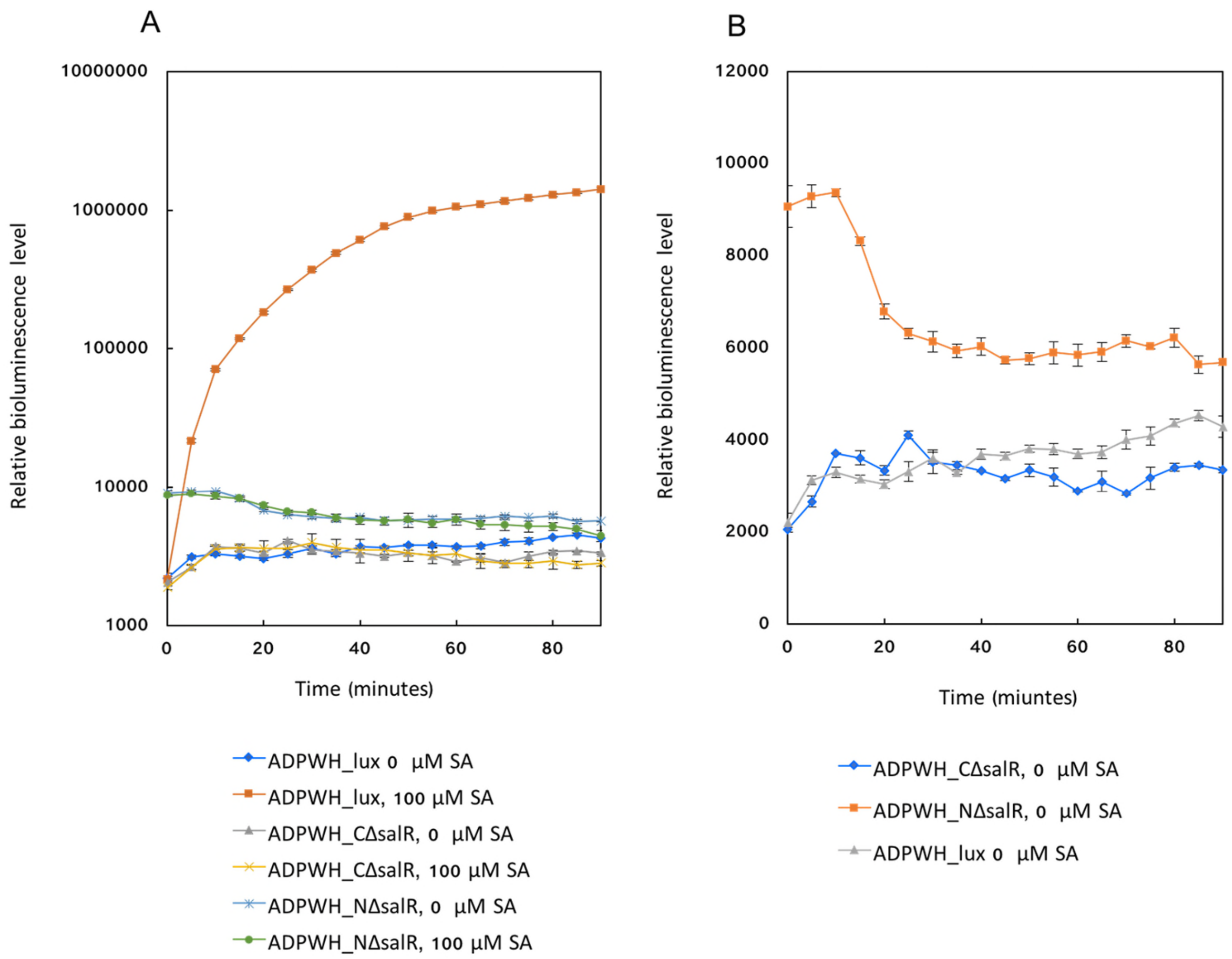

Figure S3. A) Bioluminescence response to SA of ADPWH_lux, ADPWH_CΔsalR and ADPWH_NΔsalR. ADPWH_lux has a strong, rapid, response to SA, while the two mutants with incomplete salR do not respond at all. Standard deviation was plot for all data replicates (n=4), while SD for some data sets were insignificant and the enlarged picture was provided in B) as a clear indication of increased background level in case of ADPWH_NΔsalR.

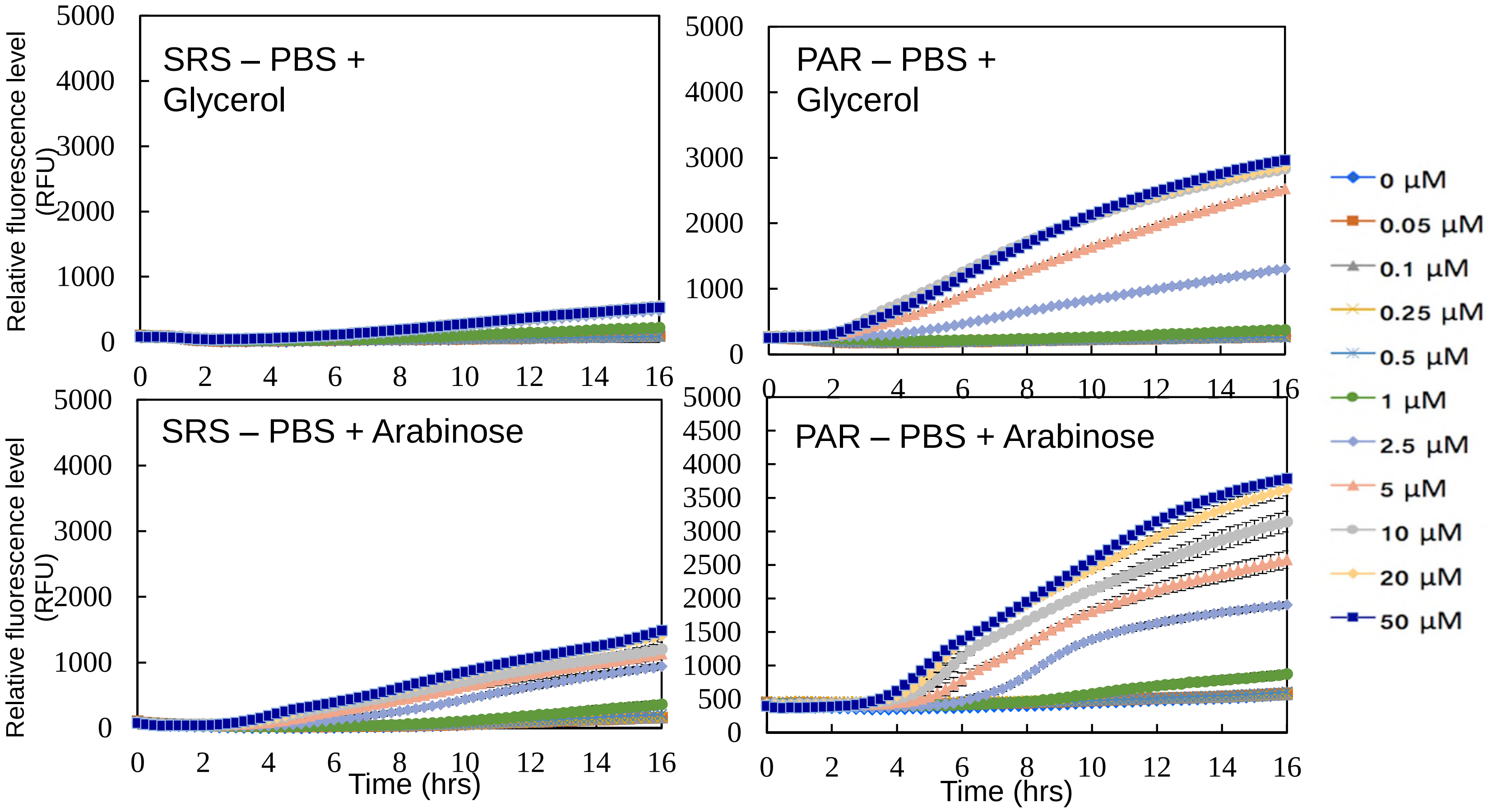

Figure S4. The effect of carbon source and growth in designed systems. Induction kinetics for the SRS and PAR configurations in (Top) PBS supplemented with glycerol (0.05%v/v) and PBS supplemented with 0.02% v/v L-arabinose (Bottom). Aspirin was added at time zero and fluorescence is observed for 16hrs. Lower panels show the *in silico* simulation result. Colour coded induction levels are indicated on the right legend. The induction gradient is 0, 0.05, 0.1, 0.25, 0.5, 1, 2.5, 5, 10, 20, 50 μM. Standard deviation was plot for all data replicates (n=4).

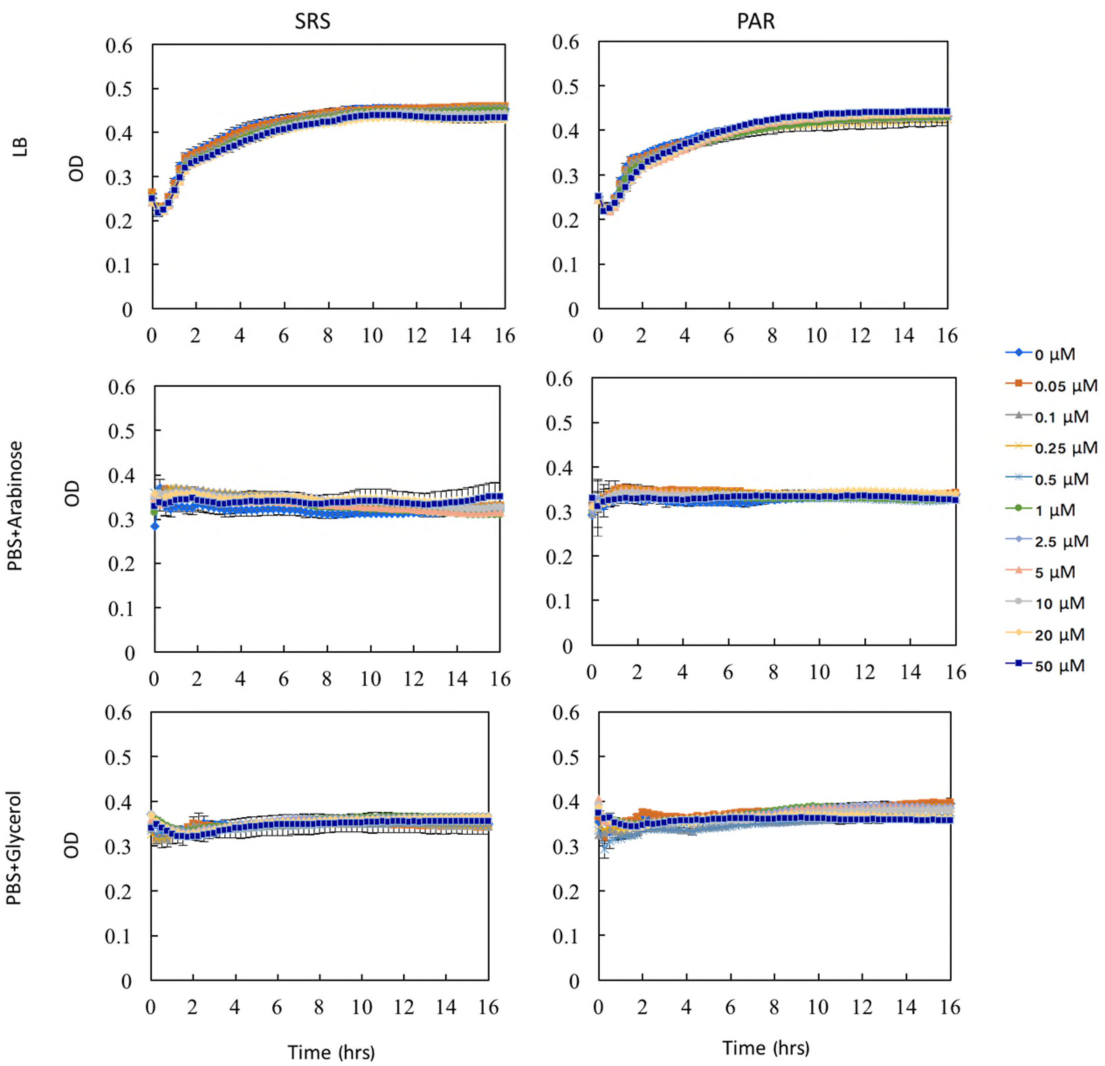

Figure S5. Growth curves of the SRS and PAR circuits in PBS supplemented with 0.02% v/v L-arabinose, PBS supplemented with 0.05% v/v glycerol. Top row illustrates that both the SRS and PAR circuits have similar growth from OD 0.2 to OD 0.42 over 16 hrs. The middle row and bottom row (PBS supplemented with arabinose and glycerol respectively) indicate that both strains have no/minor growth over 16hrs. Standard deviation was plot for all data replicates (n=4).

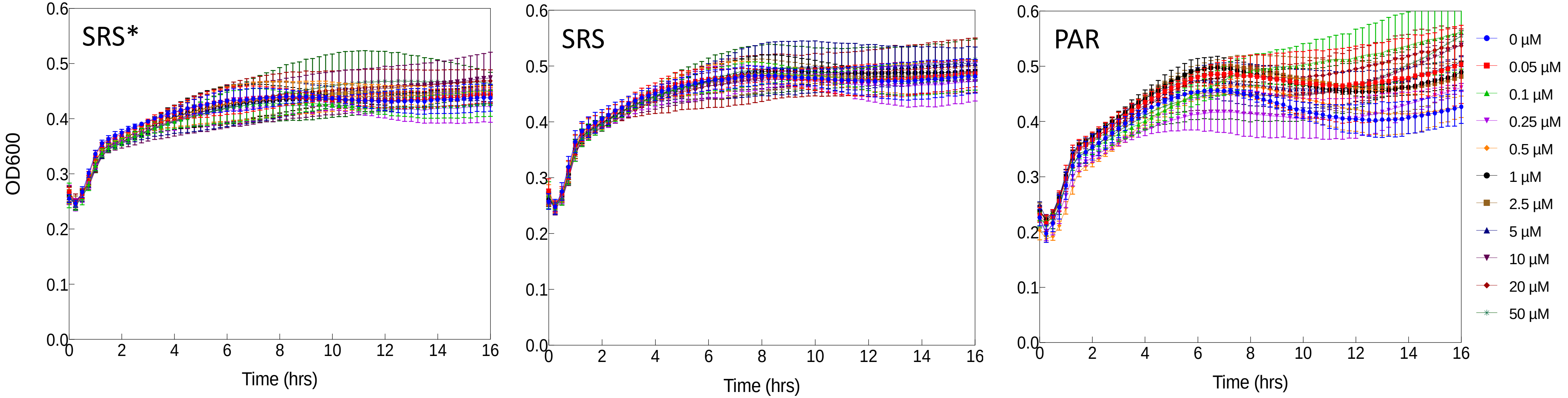

Figure S6. Growth kinetics for the SRS SRS* and PAR circuits in EcN under micro-aerobic condition and nutrient rich medium (LB). Standard deviation was plot for all data replicates (n=3).

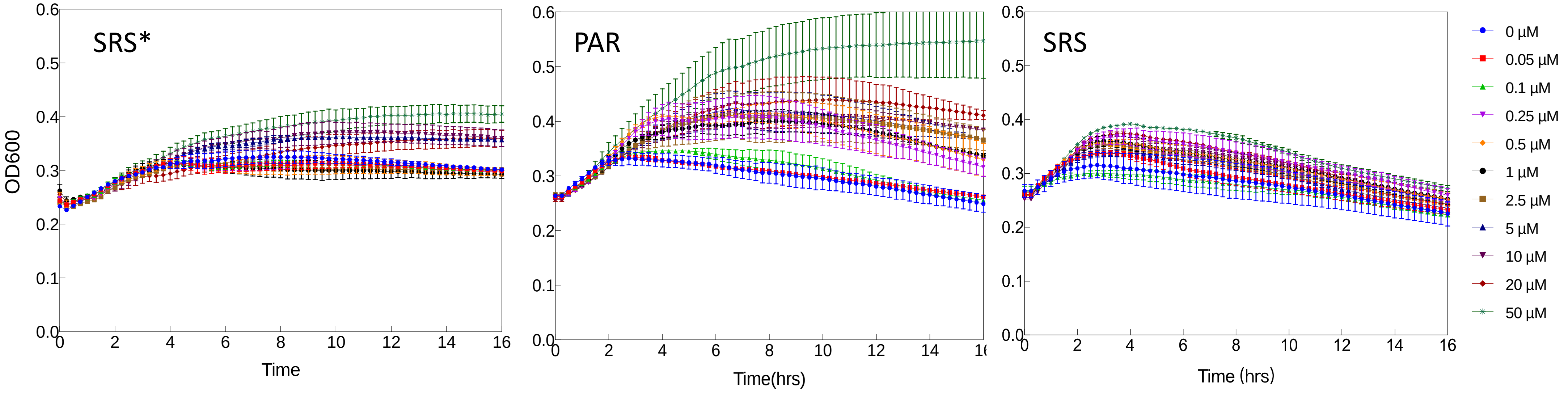

Figure S7 . Growth kinetics for the SRS SRS* and PAR circuits in EcN under micro-aerobic and nutrient stringent medium (M9 supplemented with glucose 0.4%w/v). Standard deviation was plot for all data replicates (n=3).

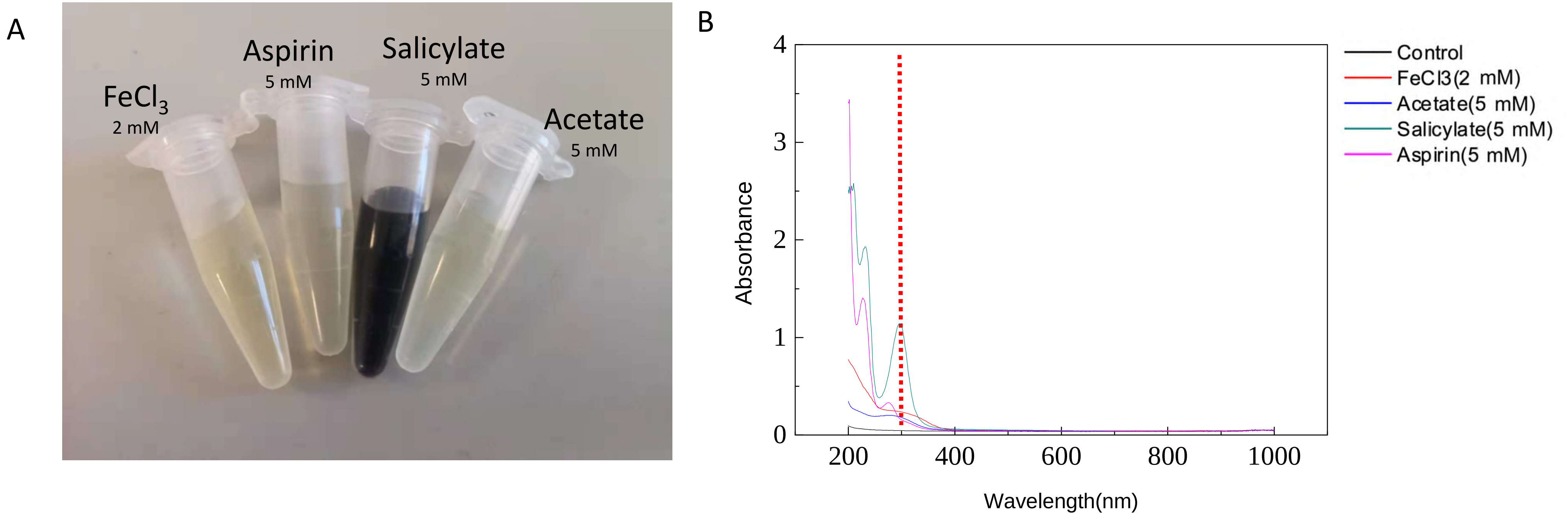

Figure S8. Fe(III)Cl_3 assay for identification of salicylate. A) Titration of 2mM $FeCl_3$ with 5 mM of Aspirin, Salicylate and Acetate respectively, only salicylate react with $FeCl_3$ yielding a dark purple color. B) Unique absorbance peak at 300nm produced from products of salicylate and $FeCl_3$. Interaction.

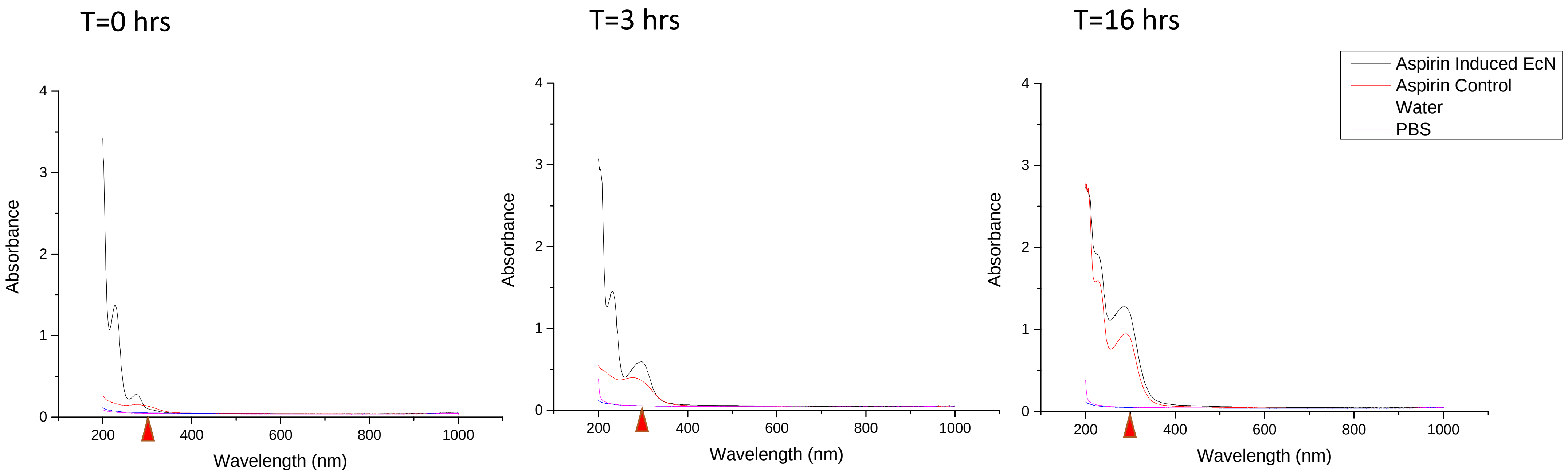

Figure S9. Aspirin conversion into salicylate. Cell supernatants were collected at time point T=0, 3 and 16 hrs after the initial aspirin induction of cells. Aspirin added into PBS medium without cells and went through the same condition as a control.

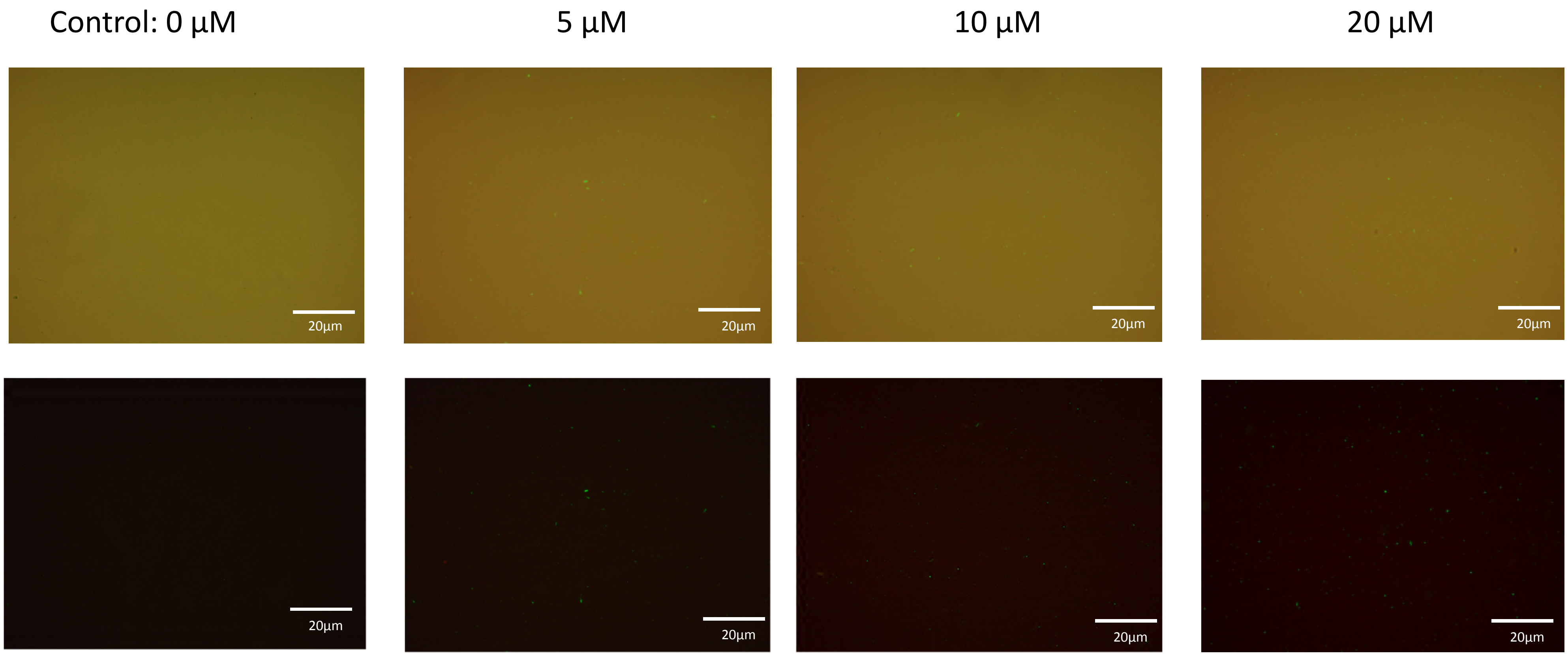

Figure S10. Microscope images of PAR_Amp* transformed SimCells. Images were taken at both GFP channel and overlaid channel for indication of potential mutated circuits or non-transformed Simcells. The specific concentration of aspirin used is indicated above plot. Background was filtered using ImagJ.
